# Landscape Genomic Analyses of *Quercus agrifolia* Née Predict Patterns of Adaptedness to Future Climate and Provide Guidance for Conservation

**DOI:** 10.1111/eva.70224

**Published:** 2026-04-21

**Authors:** Ryan C. Buck, H. Scott Butterfield, Elizabeth Hiroyasu, Jeanette Howard, John Knapp, Zachary Principe, Victoria L. Sork

**Affiliations:** ^1^ Department of Ecology and Evolutionary Biology University of California Los Angeles California USA; ^2^ La Kretz Center for California Conservation Sciences University of California Los Angeles California USA; ^3^ The Nature Conservancy, California Program Sacramento California USA; ^4^ Catalina Island Conservancy Avalon California USA; ^5^ Institute of the Environment and Sustainability, University of California Los Angeles California USA

**Keywords:** coast live oak, conservation genomics, land management, landscape genomics, oak ecosystems, *Quercus agrifolia*, restoration

## Abstract

Habitat destruction and climate change are two primary drivers of global biodiversity loss. Many contemporary ecological preserves and preserve networks are designed to protect species and the ecological and evolutionary processes that will maintain future biodiversity. However, it is challenging to identify where to locate preserves and how to manage them to meet these goals. One emerging conservation tool is landscape genomics, which allows us to identify populations that may be maladapted to future climate and thereby helps prioritize areas for acquisition (areas of high adaptedness), restoration (areas of low adaptedness), and management. Here, using whole genome sequence data of 171 adult trees, we studied the climate adaptedness of California populations of coast live oak ( *Quercus agrifolia* ), a foundation species that broadly supports biodiversity, to develop a conservation management strategy, including direct acquisition, land management, and restoration that addresses the predicted impact of future climate. Over the range of coast live oak, we find that the northernmost and southernmost stands are predicted to be more at risk for maladaptation to climate change, with the central coast of California containing stands with high adaptedness, including in unprotected areas that might benefit from protection. Using three large preserves (focal sites) managed by The Nature Conservancy (TNC), we illustrate how moving seeds from low to high elevation parts of preserves could benefit future populations range‐wide by increasing overall climate adaptedness. We discuss the different levels of risk across focal sites and apply our interpretations into TNC's 400,000 + −acre conservation holdings in California to sustain future populations of this foundational tree species. This study represents a unique collaboration between academic and applied conservation scientists to both assess levels of maladaptedness of a foundational tree to predicted climate change and also develop a genomic‐informed seed transfer program to improve adaptedness of future populations.

## 1 Introduction

Anthropogenic disturbances, such as climate change and habitat destruction, have altered ecosystems globally and are two of the primary drivers of biodiversity loss (Parmesan and Yohe 2003; Tylianakis et al. 2008; Bellard et al. 2012; Pecl et al. 2017). Predicted rapid climate change—and the uncertainties it brings for both private and public land protection and management—is forcing land managers to consider conservation strategies that maintain ecological and evolutionary processes, protect multiple species—especially those with foundational or keystone roles—and account for species' responses to future climate scenarios (Christensen et al. 1996; Lawler et al. 2010; Groves et al. 2002). Directing resources towards the protection and restoration of foundation species can indirectly preserve whole ecosystems. Foundation species, such as trees, create, and define entire ecosystems by connecting species through structural and functional support, regulating nutrient fluxes, hydrology, food webs, and biodiversity (Ellison et al. 2005; Ellison 2019). Due to their abundance, foundation species can undergo considerable declines without appearing to be in jeopardy, yet even those reductions in abundance can have cascading impacts on ecosystems across vast swaths of geographical areas (Gaston and Fuller 2008). Thus, their support of ecosystem structure and processes may deteriorate long before the species itself disappears (Ellison et al. 2005). It is therefore imperative to protect foundation species before they decline below levels needed to maintain ecosystem function because the interaction networks they support are difficult to recover and can cascade into entire ecosystem collapse (Valiente‐Banuet et al. 2014; Ellison 2019).

Genomic data provide crucial information for foundation species by identifying populations with potential high and low adaptedness, or the degree to which an organism is able to live and reproduce in a given environment (sensu Allard 1988), to future climate scenarios through landscape genomic approaches (Sork et al. 2013). Utilizing landscape genomic methods, one can examine adaptive variation across species distributions to model the associations between allelic and environmental gradients and predict the genomic composition necessary for future climate adaptation (Fitzpatrick and Keller 2015; Ingvarsson and Bernhardsson 2020; Fitzpatrick et al. 2021; Rellstab et al. 2016, 2021). The difference between the future composition needed and the current composition is termed genetic offset (Fitzpatrick and Keller 2015). It is a measure of how much evolutionary change will be required to maintain adaptation to a future environment and can be used to identify populations in a species' range that are at the greatest risk of fitness decline (maladaptation) (Capblancq et al. 2020; Gougherty et al. 2021; Rellstab et al. 2021). We posit here that individuals with low offset are deemed more preadapted to future climate and have higher relative “adaptedness.” Rather than focusing on the “maladaptedness” of individuals, which is measured by the degree of genomic offset, we use an adaptedness index, calculated here as −1 × genomic offset, wherein larger values indicate a higher degree of adaptedness and smaller values indicate a lower degree. We point out that the adaptedness index is directly related to genomic offset but its use provides a better description of the topic under consideration. Many tree studies have used genomic offset to locate populations predicted to be maladapted under climate change and to identify seed sources for assisted dispersal (Martins et al. 2018; Varas‐Myrik et al. 2022; Lachmuth et al. 2023; Yuan et al. 2023; Mead et al. 2024), where seeds from preadapted populations are used to increase the adaptedness of populations predicted to be maladapted. Using these adaptive variation‐based metrics to identify seed sources may be more predictive than climate distance alone and more pragmatic than long‐term common garden experiments, an essential alternative for slow growing species (Fitzpatrick et al. 2021; Yu et al. 2022; Lachmuth et al. 2024). For example, Mead et al. (2024) found that selecting seed sources based on genomic data rather than focusing on using only local seeds may increase the adaptedness of island oak ( *Quercus tomentella* ) populations to climate change across California's Channel Islands. While the number of landscape genomic studies is increasing (Dauphin et al. 2023), few have applied these methods to provide practical solutions for land managers and conservation practitioners (but see Mead et al. 2024).

Here, we explore the landscape genomics of a foundation tree species endemic to California, coast live oak ( *Q. agrifolia* ), which covers ~1.5 million acres across California with 40% of that located in lands with some form of protection status. Even with a large proportion of its populations in protected habitat, coast live oak is threatened by insect infestations, including from the goldspotted oak borer ( *Agrilus auroguttatus* ) (Coleman and Seybold 2009; Coleman et al. 2011), diseases, such as sudden oak death (caused by *Phytophthora ramorum*) (Swiecki and Bernhardt 2015), and incompatible cattle grazing (Snow 1972; López‐Sánchez et al. 2014). Ecological niche models predict an overall increase in coast live oak's distribution in California by 2070 (Ramírez‐Preciado et al. 2019), but it is not known whether the existing trees will be maladapted to future climates or whether stands in protected lands hold enough preadapted genotypes to persist. Our overarching goal here is to demonstrate how assessing the adaptedness of a foundation species through landscape genomics can be implemented into conservation management practices to preserve entire ecosystems in a changing climate.

This study was undertaken in collaboration with The Nature Conservancy (TNC), one of the largest private oak woodland landholders in California. Here we utilize access to TNC's more than 400,000‐acre grassland and oak woodland land holdings in California (Figure S1) to evaluate the status of coast live oak and to inform potential seed transfer designs for management and restoration of coast live oak populations for a resilient future. This distinctive partnership presents a unique opportunity to not only examine climate adaptedness but also directly apply our findings into management recommendations across large portions of coast live oak habitat, thereby encouraging additional management across the state. Specifically, our objectives are to: (1) assess the adaptedness of coast live oak stands, (2) determine if protected lands harbor stands with higher adaptedness than unprotected lands, and (3) illustrate the use of genomics in conservation by developing strategies for three TNC preserves (focal sites) to increase their stands' adaptedness. This research provides a compelling case study in the application of landscape genomic analyses for practical solutions to maintain and restore climate‐adapted ecosystems.

## 2 Methods

### 2.1 Study Species

**FIGURE 1:**
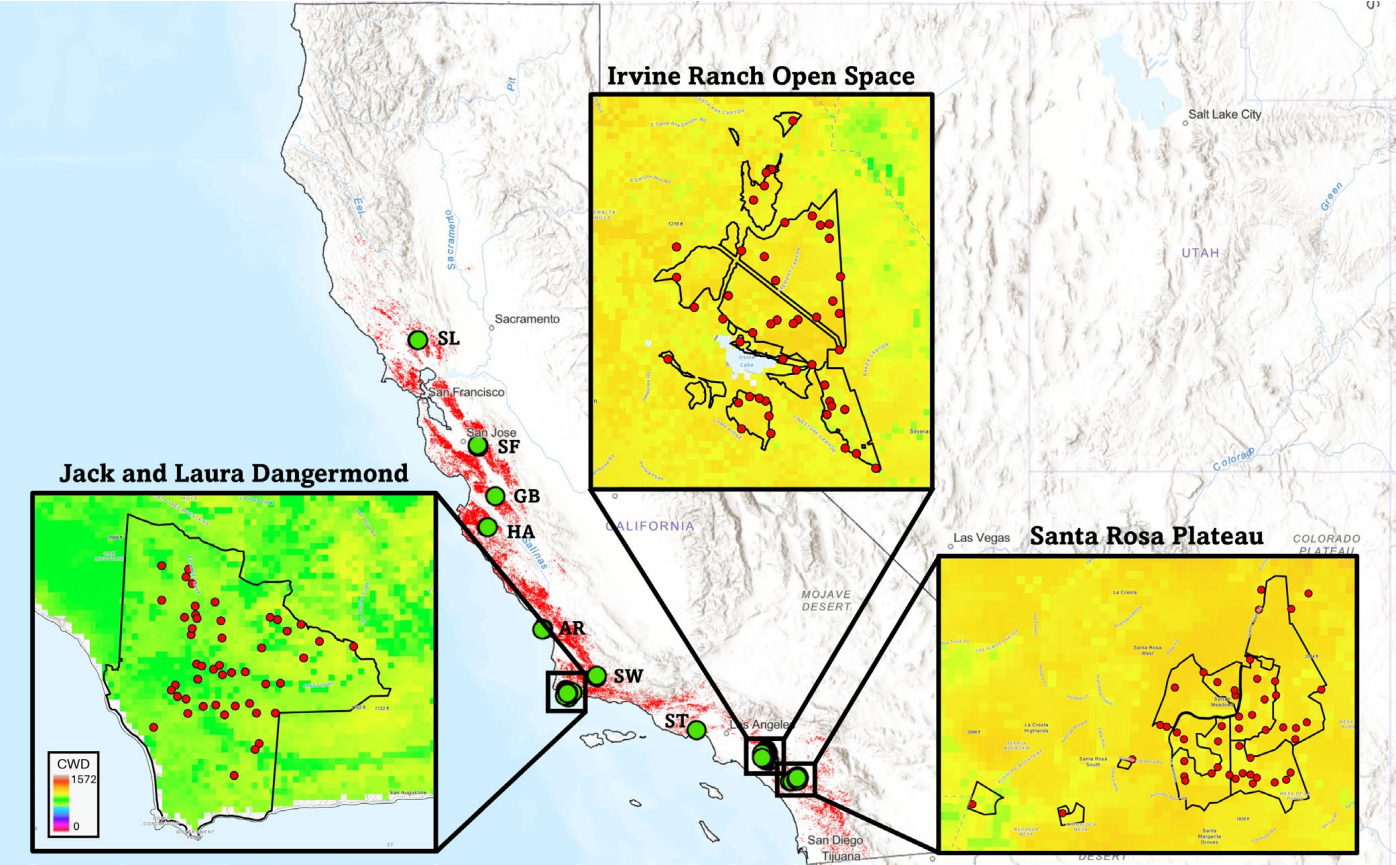
Sampling map of coast live oak (full distribution in red), with sampling sites represented as green circles and TNC focal sites encompassed by squares. Overlaid images are enlarged maps of the focal sites, with red dots representing sampled individuals, and the climate water deficit variable as the background, with warmer colors indicating higher deficit.

Coast live oak is a native, drought‐resistant, evergreen tree. Classified as a red oak (*section Lobatae*), it occurs in the coastal ranges from Mendocino County, California to Canada El Piquillo, Baja California, Mexico (Steinberg 2002; Figure 1). Stands are generally between 40 and 110 years old (Pillsbury and Joseph 1991), but individual trees may live for well over 250 years (Plumb and Gomez 1983). Typically, coast live oak forms oak woodlands that intersperse with open grassland, which can differ in species composition, biomass, and species richness (Parker and Muller 1982; Marañón and Bartolome 1994). It can co‐occur with several other oak species including black oak ( *Q. kelloggii* ), blue oak ( *Q. douglasii* ), canyon live oak ( *Q. chrysolepis* ), Engelmann oak ( *Q. engelmannii* ), interior live oak ( *Q. wislizenii* ), and valley oak ( *Q. lobata* ). Coast live oak appears to be more susceptible to seedling browsing damage (Griffin 1971; Muick 1991; Dunning et al. 2002), but more drought tolerant (Chacon et al. 2020) and fire resistant (Steinberg 2002) than some Californian oaks.

### 2.2 Sample Collection and Range Extrapolation

In June 2023, multiple leaves from 171 mature coast live oak individuals were collected, immediately stored in ice, and transported to an ultracold freezer (−80°C) at UCLA for future analyses. The sampled trees were distributed across 10 sampling sites, including preserved areas managed by TNC, the University of California Reserve System, Orange and Riverside County Parks, California Department of Fish and Wildlife, and California State Parks (Table 1), with more intensive sampling in three TNC focal sites dispersed across the state: the Jack and Laura Dangermond Preserve (The Nature Conservancy 2017; Butterfield et al. 2019), the Irvine Ranch Open Space, and the Santa Rosa Plateau (Figure 1). This individual‐based sampling design captures the environmental and genetic variation across the range of coast live oak (Figure S2) and also includes a finer resolution of gradients within focal sites. Within each focal site, a random stratified sampling design was employed, where each site was broken into 2 km × 2 km grids and a single point was randomly added to each grid, ensuring that each point occurred within the known range of coast live oak, was > 200 m apart, and within 500 m from an access road. Points were mapped using satellite data and placed on the nearest tree. The current range of coast live oak was assembled using occurrence data from CALVEG (USDA Forest Service 2018) and VegCAMP (California Department of Fish and Wildlife 2024).

**TABLE 1:** Sampling sites of coast live oak (CLO) across the species range, with approximate coordinates of each site.

Site	Land type	Preserve area (acres)	CLO cover (%)	Approximate coordinates	Trees collected
Sugarloaf State Park (SL)	State Park	4697	11	38.441, −122.522	4
San Felipe Ranch (SF)	TNC easement	28,136	31	37.275, −121.683	4
Gabilan Ranch (GB)	TNC easement	11,021	41	36.729, −121.449	4
Hastings Reserve (HA)	UC Reserve	2373	70	36.382, −121.559	3
Andre Ranch (AR)	TNC easement	1721	95	35.242, −120.778	4
Sedgwick Reserve (SW)	UC Reserve	5896	35	34.695, −120.042	4
Jack and Laura Dangermond Preserve^a^	TNC fee	24,337	34	34.532, −120.459	48
Stunt Ranch (ST)	UC Reserve	310	8	34.092, −118.657	4
Irvine Ranch Open Space^a^	TNC easement^b^	11,379	4	33.869, −117.692	48
Santa Rosa Plateau^a^	TNC co‐managed^c^	5863	26	33.516, −117.309	48

^a^ TNC focal site where more intensive sampling was done.

^b^ Owned by Orange County Parks.

^c^ Owned by the California Department of Fish and Wildlife and Riverside County Parks.

Protected lands were extracted from the California Conservation Easement Database (CCED 2024), which includes lands protected under conservation easements, and the California Protected Areas Database (CPAD 2024), which includes lands owned in fee and protected for open space purposes by over 1000 public agencies and non‐profit organizations. All geofiles were mapped and merged in ArcGIS Pro (ESRI 2025).

### 2.3 DNA Extraction and Sequencing

Approximately 50 mg of leaf tissue from each sample was flash‐frozen in liquid nitrogen before bead grinding. DNA was then extracted using a modified version of the Qiagen DNeasy Plant Mini Kit protocol preceded by a prewash step, following Mead et al. (2024) and Li et al. (2007). The prewash buffer, consisting of 100μL of Tris, 100μL of EDTA, 200μL of 5 M NaCl, 600μL of molecular grade water, and 0.01 g of PVP, was applied twice to each sample to remove polyphenols. Extracted DNA was then sent to UC Davis DNA Technologies and Expression Analysis Core Laboratory for library preparation using a custom seqWell kit and whole genome sequencing (WGS) on a NovaSeq 6000 150 bp paired‐end sequencer.

### 2.4 Filtering and Variant Calling

To produce a whole genome dataset, adapter sequences were trimmed from raw reads using Trim Galore, removing reads less than 20 bp in length. Reads were then aligned to the most closely related publicly available chromosome‐level reference genome, *Q. rubra* (Kapoor et al. 2023), using BWA‐MEM (Li 2013). Duplicate reads were marked and removed using the MarkDuplicates tool in GATK (der Van Auwera and O'Connor 2020). Variants were called using the “HaplotypeCaller” tool in GATK with the “emit‐ref‐confidence” option set to “GVCF”. GVCFs were then imported into GenomicsDB via GATK GenomicsDBImport and genotyped using “GenotypeGVCFs”. Variants were hard‐filtered using the “VariantFiltration” tool in GATK, with SNPs and indels filtered separately. For SNPs, variants with quality by depth (QD) < 2, quality (QUAL) < 30, mapping quality (MQ) < 40, phred‐scaled strand bias (FS) > 60, symmetric odds ratio strand bias (SOR) > 3, mapping quality rank sum (MQRankSum) < −12.5, and read position rank sum (ReadPosRankSum) < −8 were removed (Mead et al. 2024). Indels with QD < 2, FS > 200, QUAL< 30, and ReadPosRankSum < −20 were removed (Mead et al. 2024). Repetitive regions of the genome were removed using vcftools based on the reference genome. From this set of high‐quality variants, biallelic SNPs with high coverage across all samples were selected for further analysis. Using vcftools (version 1.15.1, Danecek et al. 2011), only biallelic SNPs were selected, individual genotypes with depth < 5 were set to missing, and SNPs with a mean depth across all samples < 5, with a minor allele frequency < 0.01, and with ≥ 10% missingness across all individuals were removed. The resulting filtered VCF file was converted to BED file format using PLINK version 1.90b6.26 (Chang et al. 2015), and variants were pruned for linkage disequilibrium using a window size of 50 variants, a window shift value of 10, and an *R* ^2^ threshold of 0.1. Genetic structure analyses were run on this filtered and LD‐pruned dataset of 948,386 SNPs. For analyses requiring no missing data, SNPs were imputed by assigning missing individuals the most common SNP (total missingness in the filtered dataset was < 7%).

### 2.5 Climate Data

Ten recent and future bioclimatic and hydrological variables (Table S1) were extracted at 270‐m resolution from the Basin Characterization Model (Flint et al. 2021; Stern et al. 2024). Variables from the 30‐year averages of 1951–1980 were selected to more closely represent the historical conditions that the sampled mature trees grew in, noting that most sampled trees were likely established 100–250 years ago based on visual height and canopy size estimates. Future bioclimatic and hydrological variables were extracted from two climate models (CNRM‐CM5: warm‐wet, HadGEM2‐ES: hot‐dry), two representative concentration pathways (RCPs) (4.5: emission reduction and 8.5: emission increase), and 30‐year averages of two time periods (2040–2069 and 2070–2099). Correlations between each pair of climate variables were calculated using the “pairs.panel” function in the R package *psych* (Revelle and Revelle 2015), removing those that were highly correlated (*R* ^2^ > 0.7) with another more derived variable to reduce the potential for false positives in genotype‐environment analyses (Capblancq et al. 2020). The final reduced set consisted of six bioclimatic and hydrologic variables: climate water deficit (cwd)—annual evaporative demand that exceeds available water, recharge (rch)—annual amount of water stored in soil, runoff (run)—annual amount of water that becomes streamflow, soil water storage (str)—annual amount of water stored in soil, minimum air temperature (tmn), and maximum air temperature (tmx).

### 2.6 Genetic Structure and Variation

To identify possible geographic barriers to gene flow and genetic differences between populations, the genetic structure of coast live oak was examined across its range and within the three focal sites. First, a principal components analysis (PCA) was performed via R package *vegan* (Oksanen et al. 2024) to assess the distribution of genetic variation. Any outlier samples revealed at this stage would likely indicate the presence of additional species or hybrids and would have been removed before proceeding. To estimate the most likely number of discrete genetic clusters (*K*) within the range and calculate ancestry coefficients, ADMIXTURE, a maximum likelihood method (Alexander et al. 2009), was performed. *K* values from 1 to 10 were tested, using the lowest cross‐validation (CV) error to select the best *K*. Ancestry coefficients (*Q*‐values) were visualized using the R package *pophelper* (Francis 2017). To test whether genetic gradients are a result of geographic distance, isolation by distance was calculated between each individual genotype using a Mantel test (Mantel 1967) in *vegan* where genetic distance was calculated as the Euclidean distance between genotypes, and geographic distance was calculated as Haversine distances using the “distHaversine” function in the *geosphere* package (Hijmans 2024). To test whether genetic gradients are a result of environmental distance while accounting for geography, isolation by environment was calculated between each individual genotype using a partial Mantel test in *vegan* where genetic and geographic distance were calculated in the same way as above, and environmental distance was the Euclidean distances of the six noncollinear scaled mean environmental variables at each individual's location.

### 2.7 Genotype‐Environment Associations and Climate Adaptedness

To examine the relationship between climate and genetic variation and identify candidate adaptive SNPs associated with bioclimatic variables, a redundancy analysis (RDA) was performed with the reduced set of six noncollinear variables. As a multivariate genotype‐environment association method, RDA accounts for variation from all bioclimatic variables simultaneously and has been shown to outperform both univariate and other multivariate approaches (Forester et al. 2018). First, partial redundancy analyses (pRDAs) were implemented using the R package *vegan* to separately and jointly evaluate the contributions of climate (using the six noncollinear bioclimatic variables), geography (using latitude, longitude, latitude × longitude), and population structure (PC1) on genetic variation, summarizing each factor's influence with an ANOVA via the “anova” function in R (Table S2; Capblancq et al. 2020). A partial, constrained RDA was then performed using the bioclimatic variables conditioned on the geographic variables and population structure to identify putatively adaptive outlier loci, retaining the two constrained RDA axes that were significant by permutation tests (Table S3). Outliers were identified with *p*‐values smaller than the conservative Bonferroni‐corrected significance threshold of *α* = 0.01/*n* (where *n* is the number of loci tested) as candidate adaptive SNPs that were then used in the analyses of adaptedness. The potential function associated with each putatively adaptive SNP was then examined by utilizing the well‐annotated *Q. lobata* reference genome (ValleyOak3.2, Sork et al. 2022) (the *Q. rubra* genome is only annotated with hypothetical proteins). Annotations from the *Q. lobata* reference genome were lifted to the *Q. rubra* genome using Liftoff (Shumate and Salzberg 2021) and known genes within 1kbp of each SNP were located.

To assess the current adaptive genetic variation across coast live oak's range and predict the mismatch between future bioclimatic variables and current adaptive variation, three methods, gradient forest (GF; Ellis et al. 2012; Fitzpatrick and Keller 2015), RDA (Capblancq and Forester 2021), and generalized dissimilarity models (GDMs; Ferrier et al. 2007; Fitzpatrick and Keller 2015) were implemented using the pRDA‐identified candidate adaptive loci and the six noncollinear bioclimatic variables (see Supporting Information Methods for more detail). Gradient Forest results are presented in the main text and used to calculate seed sources because several studies have begun validating their GF‐modeled predictions with common garden data and simulations (Fitzpatrick et al. 2021; Láruson et al. 2022; Lachmuth et al. 2023; Archambeau et al. 2026; Verrico et al. 2026). It is important to note that while these analyses are built on the genotypes of sampled individuals, the climate associations and resulting predictions are limited to the resolution of climate data (270 m × 270 m grid cells here). Adaptedness, calculated here as −1 × genomic offset, is therefore predicted for entire grid cells and, as such, we refer to the trees existing in those grid cells as **“stands”** because they are composed of multiple individuals. This topic is discussed further in the *Caveats to Our Study* section below.

Due to the varying model assumptions, units, and scales of the three adaptedness methods, raw adaptedness values cannot be directly compared. Differences in relative patterns of spatial variation among the three models' adaptedness predictions were thus quantified in multivariate space using Procrustes residuals (Peres‐Neto and Jackson 2001) via the “Procrustes” function in the *vegan* package and mapped using Fitzpatrick and Keller's (2015) “RGBdiffMap” function. This method allows for the relative magnitude of adaptedness values to be spatially compared among each model despite their differences. The resulting Procrustes residuals represent the degree of mismatch in adaptedness predictions at each grid cell. Additionally, to quantify concordance among adaptedness models, we calculated Spearman rank correlations (Spearman 1904) between adaptedness models for each climate scenario, RCP, and time period separately.

To determine if protected lands harbor individuals with higher adaptedness than unprotected lands, an ANOVA comparing the effects of land protection status (CPAD 2024; CCED 2024), climate model, RCP, and year on GF adaptedness values was performed in R using the “aov” function with all predictors treated as fixed effects, with year, RCP, and model included as nested terms to reflect the hierarchical structure of the climate scenarios (Adaptedness ~ Protection * Model/RCP/Year). A Tukey's post hoc test was used to determine significant differences among factors via the “TukeyHSD” function.

### 2.8 Seed Sourcing Analyses

To identify stands likely to include seed sources preadapted to the future climate of TNC's focal sites, the predicted adaptedness of potential seed sources if transferred to different future locations was calculated. To do this, reverse adaptedness values were calculated between each donor cell (raster grid cell that will act as a hypothetical seed source) and recipient cell (raster grid cell that will act as a hypothetical planting location with future climate). Reverse adaptedness (Figure S3A) examines the genotype‐environment discordance between the adaptive variation needed in the future climate of a recipient cell and the adaptive variation currently present in all potential donor cells within the range to determine which current donor stands contain adaptive compositions closest to what is needed in the future recipient cell (Gougherty et al. 2021). It can be used to examine potential seed sources of focal sites by examining existing stands across the species range and determining which are most preadapted to the future climate of the focal site. We first calculated reverse adaptedness using cells from across the entire coast live oak range as donors and cells within each focal site as recipients. The adaptedness values for each donor cell (cells across entire species range) were averaged across all recipient cells (cells in each focal site) to give an average reverse adaptedness value of each donor cell if transferred to an alternative site under future climate, indicating how good the donor cell would be, on average, as a seed source for the entire focal site in future climate. Using these values, we then calculated seed source priority, or the percent of the focal site that a donor cell would increase adaptedness for if used as a seed source—with the higher the percent of the focal site increased, the higher the priority of the donor as a seed source. To do this, the donors' adaptedness values after seed transfer were compared to the recipients' baseline adaptedness values (adaptedness if no seed transfer occurred) creating a binary matrix with “1” indicating the donor's adaptedness value after seed transfer is higher than the recipient's baseline adaptedness, meaning that if the donor cell is used as a seed source there is a predicted increase in adaptedness at the recipient cell and the donor is thus a better seed source, and “0” indicating the donor's adaptedness after transfer is lower than the recipient's baseline adaptedness, meaning that if the donor cell is used as a seed source, adaptedness is predicted to decrease at the recipient cell and thus the donor is a worse seed source. The binary values were then summed for each donor cell and divided by the total number of recipient cells in the focal site to give a percentage of the focal site that the donor cell would increase adaptedness for if that donor cell was used as a seed source. All raster cells within each focal site were used as recipient cells. We limited the first analysis to all cells within the focal site as donors, then extended donors to all cells within a 50 km radius of the focal site to find alternative but still relatively local seed sources, and finally expanded the analysis using cells across the entire species range as donors. This approach determines the percent of the focal site that the donor cell would increase adaptedness for and allows for the identification of the best seed sources for the entire preserve.

To identify regions of coast live oak's range to which stands in each of TNC's focal sites are likely preadapted (have high adaptedness values in) if used as seed sources, forward adaptedness values were calculated between each donor cell and recipient cell. Forward adaptedness (Figure S3B) examines the genotype‐environment discordance between the adaptive variation present in the current climate of a donor cell and the adaptive variation needed in the future climate of all potential recipient cells within the range to determine which future recipient cells the current donor stands' adaptive compositions are most preadapted to (Gougherty et al. 2021). It can be used to examine potential planting locations for individuals from our focal sites by examining the current adaptive composition of stands in focal sites and determining where in the future landscape they would be most preadapted to. We calculated forward adaptedness using cells from each focal site as donors and cells across the entire species range in future climate as recipients. The adaptedness values for each recipient cell (cells across the entire range) were averaged across donor cells (cells within the focal site) to give an average forward adaptedness of each recipient cell, indicating how good the recipient cell would be, on average, as a planting location if the entire focal site was used as a seed source.

For this study, we examine entire focal sites because no specific restoration area has been identified. From a practical point of view, land managers are likely to focus on the restoration of specific parts of their preserve, in which case it would be more prudent to match seed sources to the future climate of that specific planting location rather than the entire preserve. Doing so without an identified restoration site would result in a near infinite number of possible combinations. Here, we use entire focal sites to exemplify how these methods can be used, but both the donor and recipient areas are scalable and can be implemented on a single grid cell to range‐wide scales.

## 3 Results

### 3.1 Genetic Structure and Variation

**FIGURE 2:**
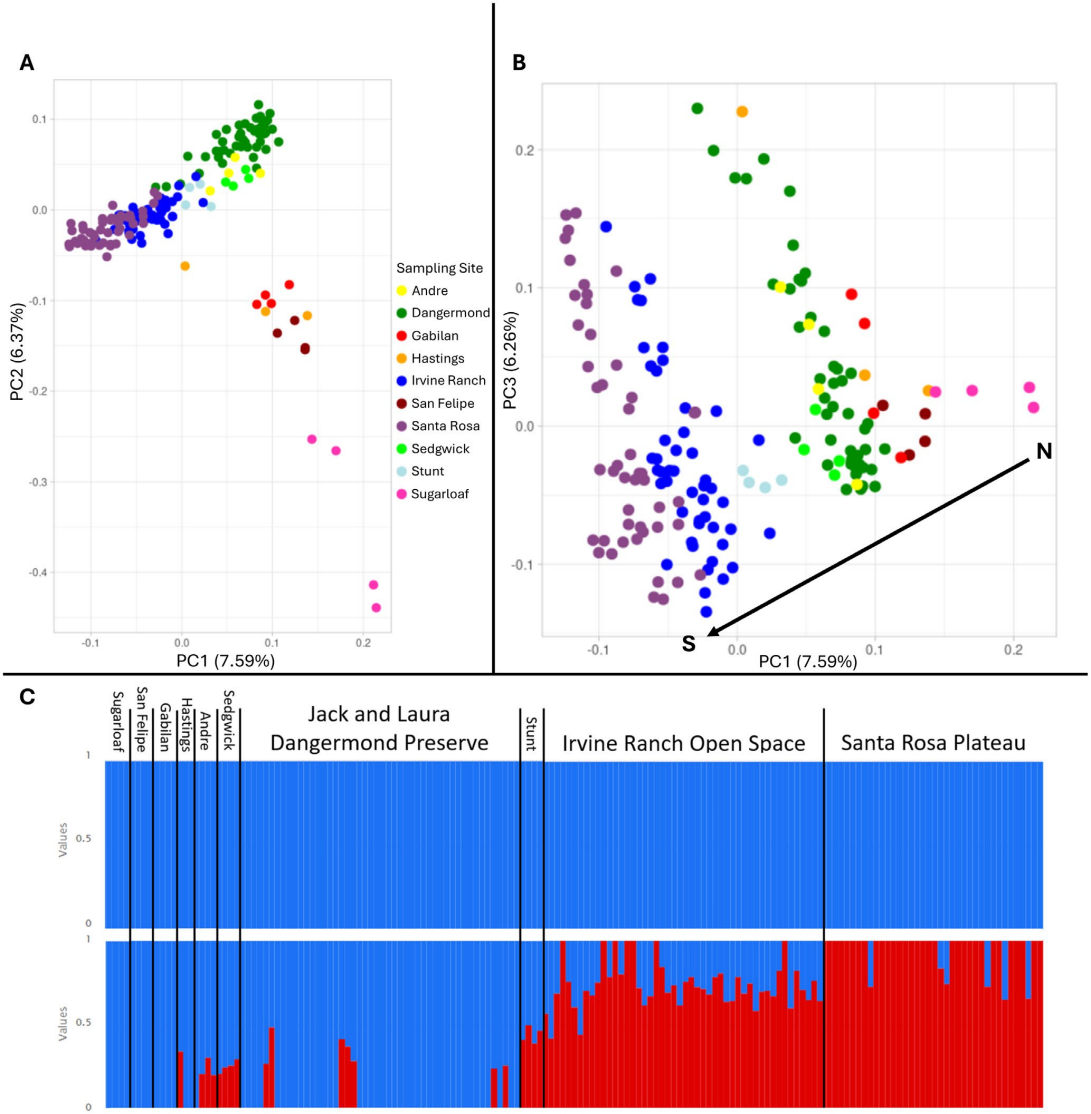
Analysis of genetic variation and structure based on whole genome sequences of 171 coast live oak adults shown in Figure 1. (A) PCA with axes 1 and 2 colored by sampling site along the color spectrum with the northernmost site being red and southernmost being purple, showing separation between the four most northern and remaining southern sites. (B) PCA with axes 1 and 3 shows genetic variation aligning with latitude. (C) ADMIXTURE plots of the same 171 adults grouped by sampling site ordered North to South, with *K* = 1–2 shown. *K* = 1 had the lowest CV error and was thus the most likely; *K* = 2 had the second lowest CV error. Each vertical bar represents an individual sample, while each color is a genetic group that individual belongs to, with multi‐colored bars representing admixed individuals based on *Q*‐values shown on the *y*‐axis.

When viewing PC axes 1 and 2 (Figure 2A), representing 7.59% and 6.37% of variation respectively, the four most northern sampling sites (Sugarloaf State Park, Gabilan Ranch, San Felipe Ranch, and Hastings Reserve) separated from the remaining six more southern sites along PC2, while PC1 spread the six southern sites North to South from right to left. When viewing PC axes 1 and 3 (Figure 2B), the latter representing 6.26% of the total variation, PC1 spread all sample sites by latitude (North to South from right to left). The PC3 axis spread points within each sampling site, exhibiting the variation present within sites. ADMIXTURE (Figure 2C) gave the best clustering (*K*) of 1 based on CV error, suggesting high gene flow, with higher values of *K* showing a trend in the genetic gradient between northern (left) and southern (right) sites. When examining isolation by distance, we found genetic distance was correlated with geographic distance, with an *R* ^2^ value of 0.045 and *p*‐value of 0.001 (Figure S4A). Similarly, PC1 was heavily correlated (*R* ^2^ > 0.8) with all three geographical variables (latitude, longitude, and latitude×longitude) (Table S4). When examining isolation by environment, we found genetic distance was not significantly correlated with environmental distance, with an *R* ^2^ value of 0.024 and a *p*‐value of 0.53 (Figure S4B).

### 3.2 Putatively Adaptive Loci and Patterns of Adaptive Diversity

The full redundancy analysis (RDA) including environment, geography, and population structure explained 7.3% of the variation in all loci (Table S2). The constrained, partial RDA conditioned on geography and population structure found that climate accounted for 49.7% of the explained variance (Table S2), identifying 1741 significant candidate adaptive SNPs. When exploring the potential function associated with each of the 1741 SNPs, 254 were located within 1kbp of a coding region, 180 of which had known functions, with 28 having associations to stress response including genes responsible for freezing tolerance, pathogen defense, growth regulation, and gene expression (Table S5).

When examining the current adaptive genetic variation across coast live oak's range, the GF models showed that the northern half of coast live oak's range had more spatial heterogeneity in the distribution of adaptive variation than observed in the southern half, with larger differences in GF adaptive composition between higher elevation coastal mountains and lower elevation inland stands (Figure S5). Within focal sites, adaptive composition was homogeneous, with little variation in adaptive diversity. When examining differences in adaptive composition across focal sites, the Jack and Laura Dangermond Preserve had slightly different adaptive composition from the Irvine Ranch Open Space and the Santa Rosa Plateau, while the Irvine Ranch Open Space and the Santa Rosa Plateau had similar composition. Maximum temperature had the highest importance of bioclimatic variables in explaining adaptive genetic variation modeled in GF, followed by climate water deficit, minimum temperature, runoff, soil storage, and finally soil recharge (Figure S6). GDM had similar importance rankings, with climate water deficit as the most important bioclimatic variable, followed by minimum temperature, soil recharge, maximum temperature, and soil storage, while runoff did not explain a significant amount of variation (Figure S7). RDA's ranking of bioclimatic variables differed the most from the other two methods, with minimum temperature ranked as most important, followed by soil recharge, climate water deficit, runoff, maximum temperature, and finally soil storage (Figure S8).

### 3.3 Adaptedness of Coast Live Oak Stands

**FIGURE 3:**
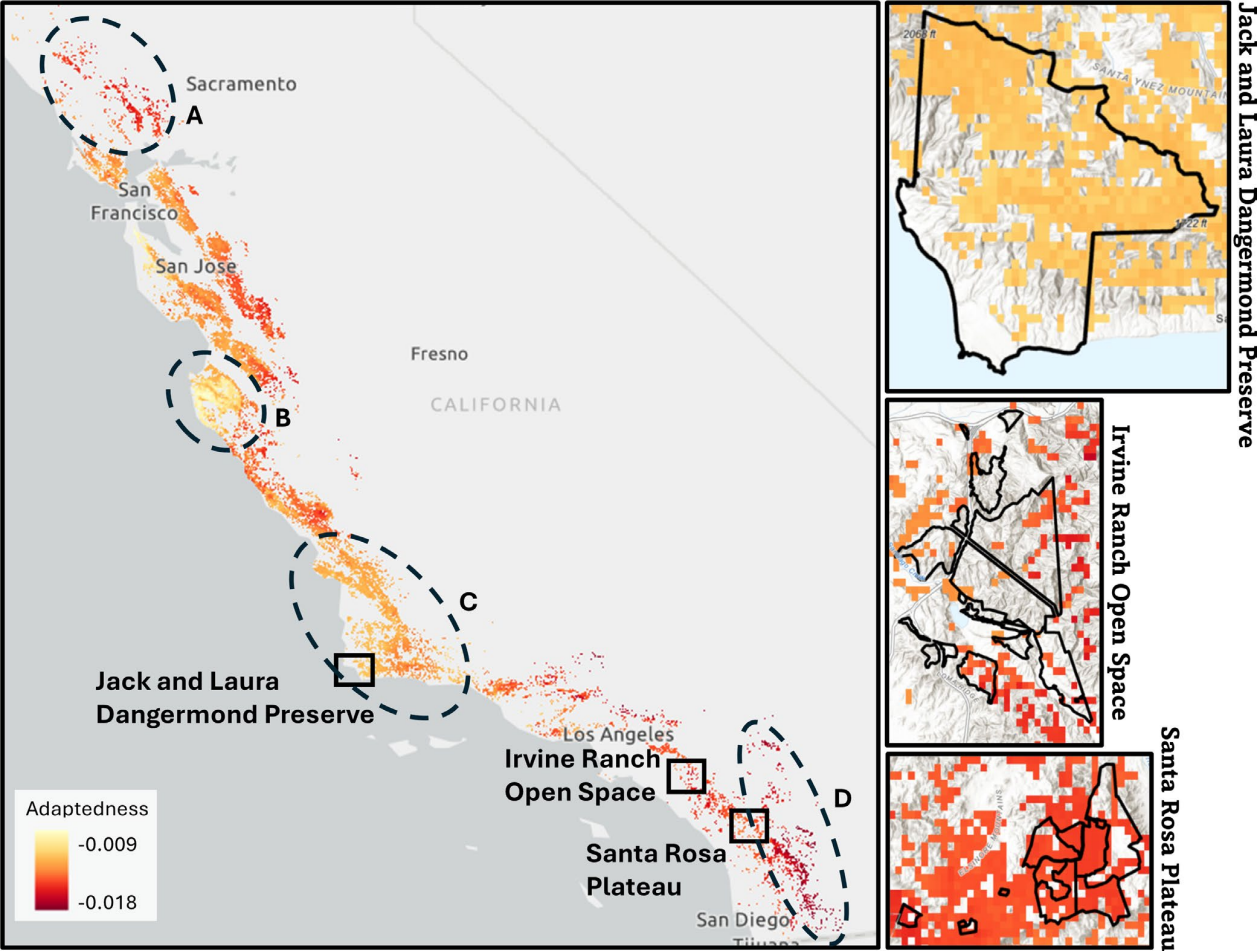
Local adaptedness predictions of coast live oak's range between recent climate and future climate model HadGEM2‐ES scenario RCP 8.5 for 2070–2099. Areas of interest with especially high or low adaptedness are circled and labeled A–D for reference within the text. Overlaid boxes contain enlarged adaptedness figures of the three TNC focal sites. Lighter colors indicate larger adaptedness values. Adaptedness values were calculated using GF.

**FIGURE 4:**
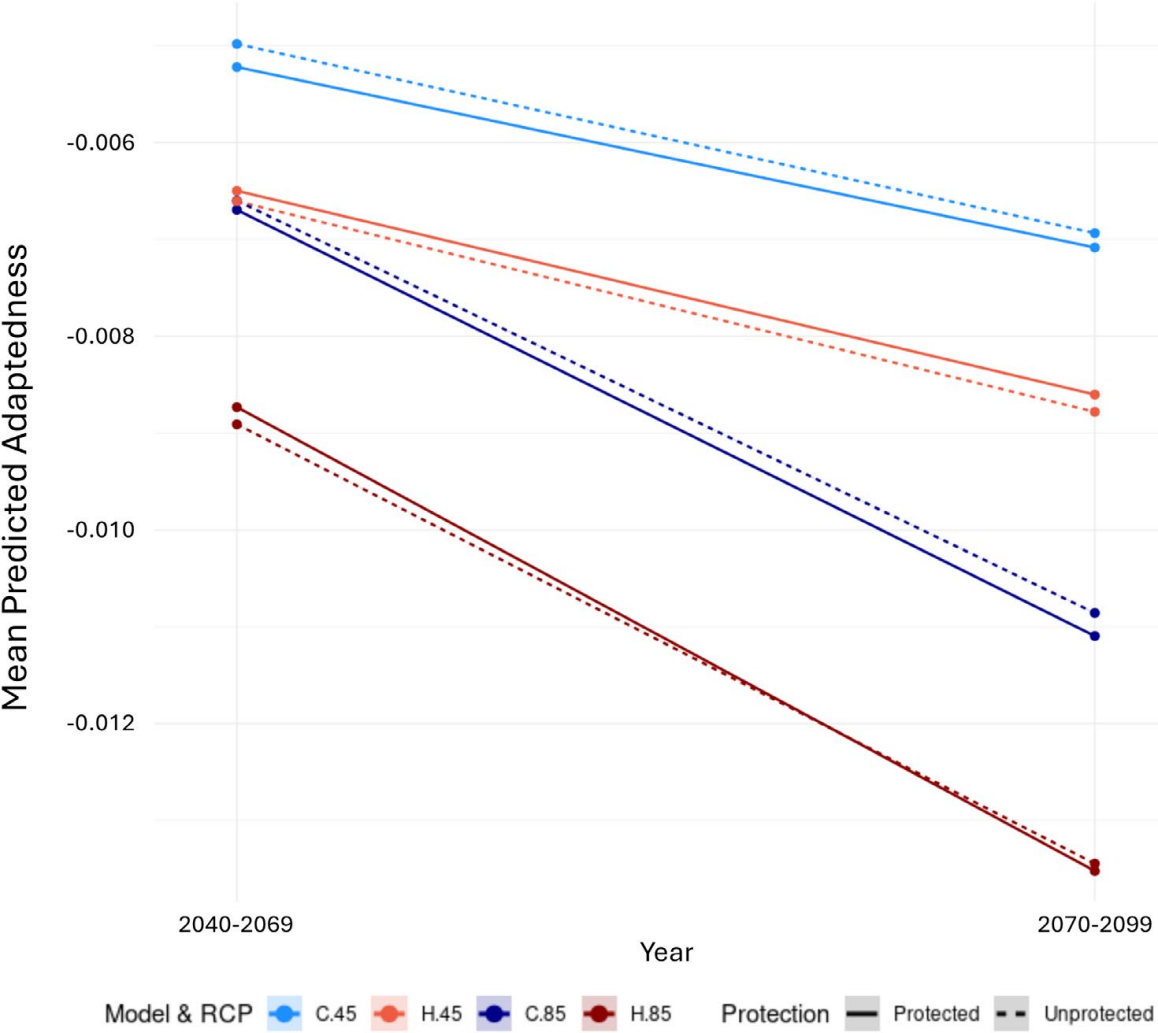
Mean adaptedness of unprotected versus protected lands across years, models, and RCPs. Average adaptedness values between CNRM‐CM5 (C, blue) and HadGEM2‐ES (H, red) for RCPs 4.5 (lighter top pair of lines) and 8.5 (darker bottom pair of lines), with line type designating protection status of land (solid line indicates protected land, dashed line indicates unprotected). All interactions are significant (*p* < 1 × 10^−15^) and all values are significantly different with the exception of adaptedness in unprotected lands in model CNRM‐CM5 RCP8.5 2040–2069 and HadGEM2‐ES RCP4.5 2040–2069.

The gradient forest models predicted a mosaic of adaptedness patterns, with stands having varying levels of adaptedness scattered throughout the coastal mountains (Figure 3, Figure S9). The lowest adaptedness values are predicted to be in stands in the northernmost (Figure 3A) and southernmost (Figure 3D) regions of coast live oak's range, specifically around Sonoma and San Diego counties. Depending on the climate model and RCP used, adaptedness in stands along the central coast of California varied in intensity, with stands with lower adaptedness predicted north of San Luis Obispo County (in between Figure 3B,C) and South of San Jose, and stands with higher adaptedness along the Central Coast, near the counties of San Francisco, Monterey (Figure 3B), and Santa Barbara (Figure 3C). On average, the CNRM‐CM5 models had significantly higher values of predicted adaptedness for coast live oak stands than the HadGEM2‐ES models (Figure 4, Figure S9), but both models showed a significant decrease in adaptedness as emissions increased (RCP 4.5 vs. RCP 8.5) and as time progressed from 2040–2069 to 2070–2100, with RCP 8.5 scenarios decreasing adaptedness more drastically (Figure 4, Table S6). The GDM and RDA models also predicted mosaics of adaptedness patterns, generally matching the predictions of the GF models with lower adaptedness predicted in stands located the northernmost and southernmost parts of coast live oak's range and patches of high adaptedness in stands along the central coast (Figures S10–S12). Procrustes residuals (Table S7) and Spearman rank correlations (Table S8) indicate similar trends in method concordance, with GDM and GF having the weakest average correlation among future climate scenarios, followed by GDM and RDA, and GF and RDA having the strongest. Visualization of Procrustes residuals (Figure S13) showed that the models differed in predictions mostly for the stands near San Jose and directly to the south along the coast, especially between GDM and GF.

When comparing protected lands to unprotected lands, adaptedness significantly varied with the protection status, model used, RCP, and year (Figure 4, Tables S9 and S10), with significant differences in adaptedness values except between unprotected lands in CNRM‐CM5 RCP8.5 2040–2069 and HadGEM2‐ES RCP4.5 2040–2069. Interestingly, coast live oak is predicted to be more preadapted to future climates in protected lands than unprotected lands under all but one (RCP8.5 2070–2099) HadGEM2‐ES models, but less preadapted (with the exception above) under CNRM‐CM5 models (Figure 4).

### 3.4 Seed Sourcing

**FIGURE 5:**
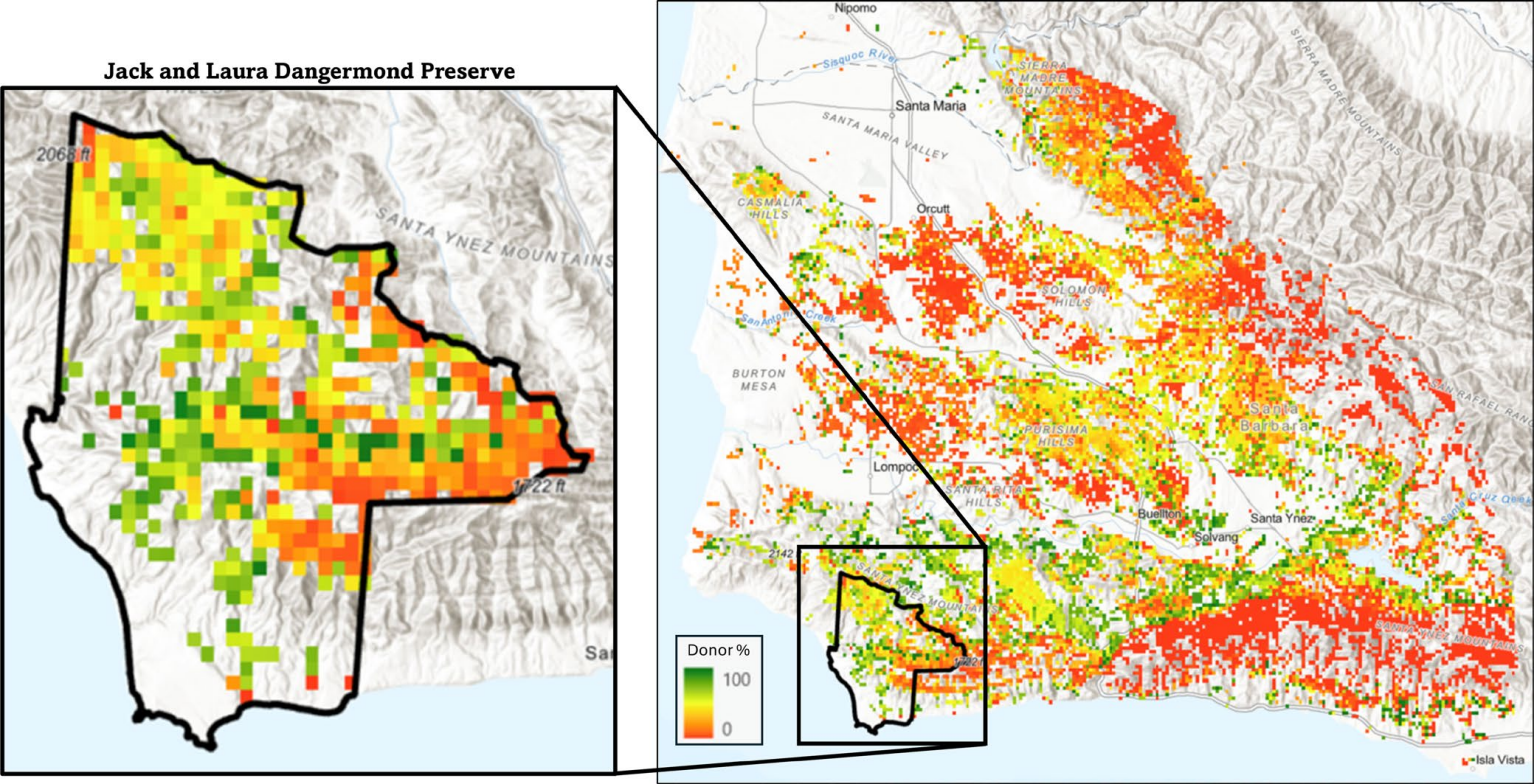
Seed source priority up to 50 km from the Jack and Laura Dangermond Preserve, modeled in HadGEM‐ES2 RCP8.5 2070‐2099, with greener colors indicating a higher percentage of the preserve that would have increased adaptedness if the grid cell was used as a seed source. The overlaid box contains enlarged seed source donor percentage within the Jack and Laura Dangermond Preserve itself. Adaptedness values were calculated using GF.

**FIGURE 6:**
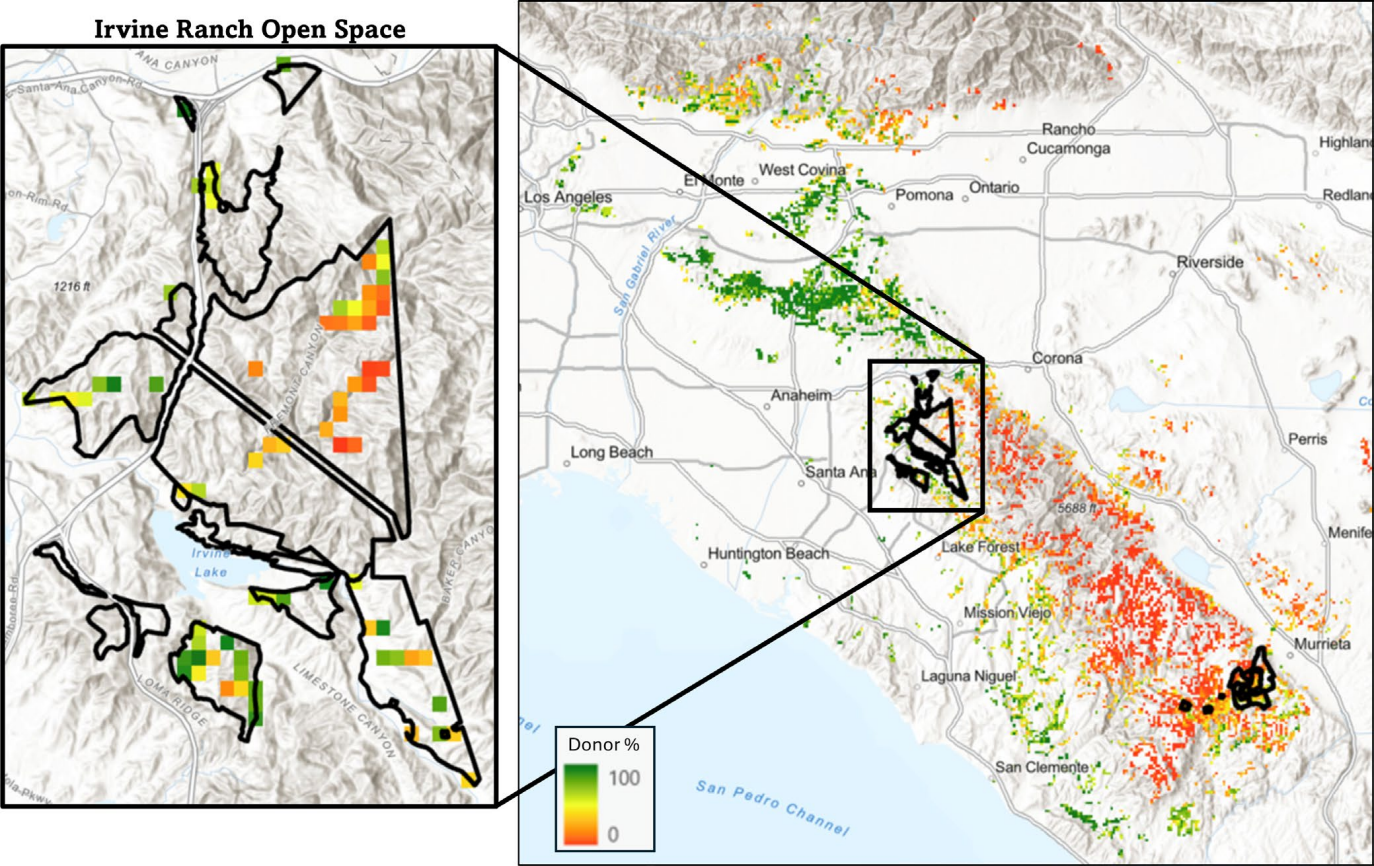
Seed source priority up to 50 km from the Irvine Ranch Open Space, modeled in HadGEM‐ES2 RCP8.5 2070‐2099, with greener colors indicating a higher percentage of the preserve that would have increased adaptedness if the grid cell was used as a seed source. The overlaid box contains enlarged seed source donor percentage within the Irvine Ranch Open Space itself. Adaptedness values were calculated using GF.

**FIGURE 7:**
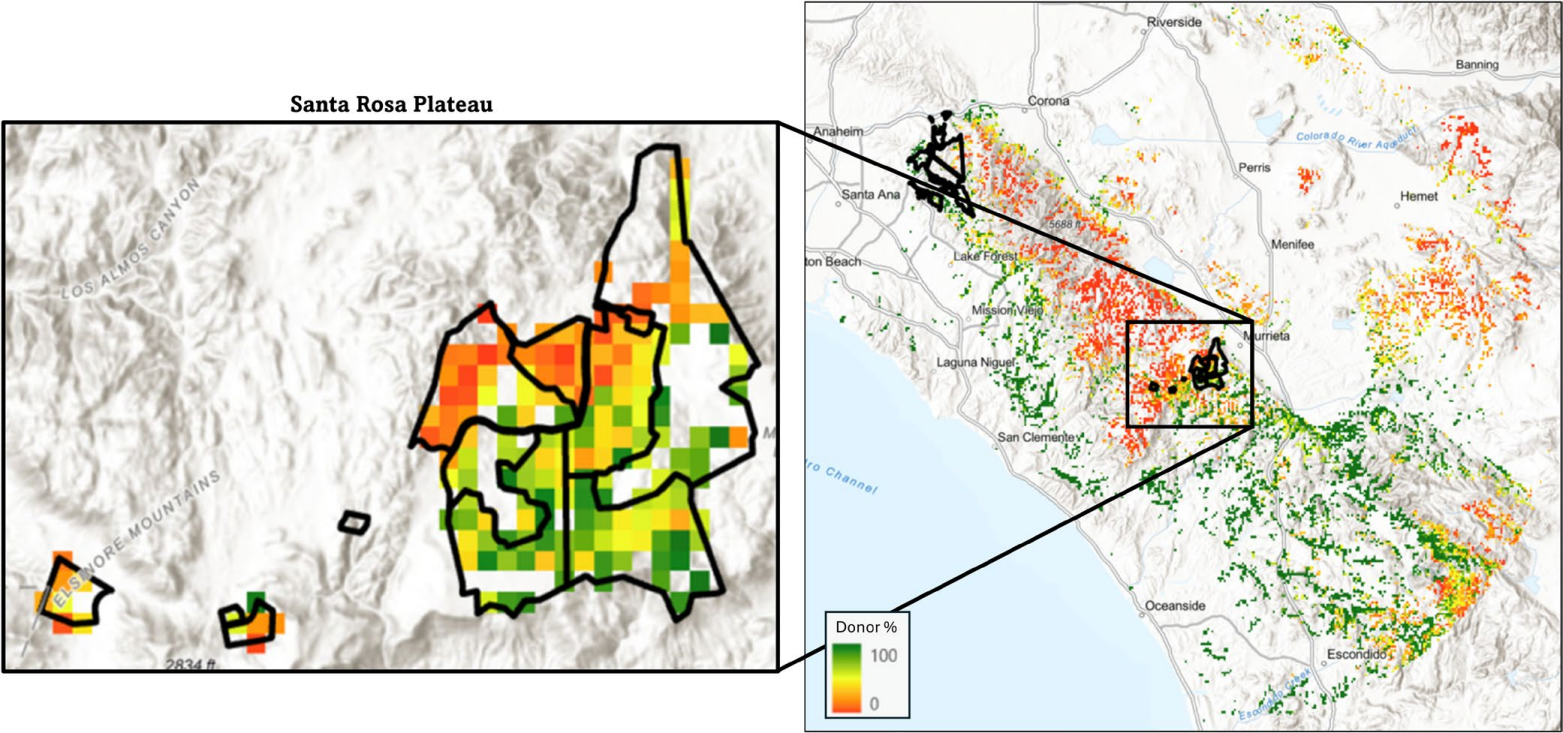
Seed source priority up to 50 km from the Santa Rosa Plateau, modeled in HadGEM‐ES2 RCP8.5 2070‐2099, with greener colors indicating a higher percentage of the preserve that would increase adaptedness if the grid cell was used as a seed source. The overlaid box contains enlarged seed source donor percentage within the Santa Rosa Plateau itself. Adaptedness values were calculated using GF.

Conservation strategies for each of the three intensely sampled focal sites are discussed below, with the potential for using seed transfer to enhance stand adaptedness in focal sites. The seed source priority maps show the percent of the focal site that each donor cell would increase adaptedness for if used as a seed source. The Jack and Laura Dangermond Preserve contains a mix of beneficial and detrimental seed sources with some sources that would, on average, increase the stands' already high adaptedness and some that would not (Figure 5, Figure S11). Generally, the lower elevation parts of the preserve, closer to the ocean, are predicted to contain better seed sources than the higher elevation more inland stands. When examining seed sources within a 50 km radius of the site, a majority of stands in the surrounding coastal mountains are not predicted to increase adaptedness, but lower elevation basins may contain better seed sources. When examining seed sources from across the entire species range, stands near San Francisco and San Jose are predicted to increase adaptedness for a majority of the Jack and Laura Dangermond Preserve's stands (Figure S14). At the Irvine Ranch Open Space, seed sources are restricted by the low tree cover within the focal site, but still include a diversity of options that would increase or decrease adaptedness (Figure 6, Figure S15). Again, higher elevation stands seem to be inferior seed sources, because they will not increase adaptedness for as many of the focal site stands as seed sources from lower elevations. When expanding the search to a 50 km radius, far more seed sources are found, however preadapted sources are restricted to lower elevation coastal facing stands in the mountains to the South and hills to the North. When examining seed sources from across the entire species range, again stands near San Francisco and San Jose, along with some stands south of Los Angeles, are predicted to increase adaptedness for a majority of stands in the Irvine Ranch Open Space (Figure S15). Within the Santa Rosa Plateau, more preadapted seed sources can be found in the southern half of the site that roughly track with elevational clines (Figure 7, Figure S16). Outside of the Santa Rosa Plateau, lower elevation stands of the coastal mountains again include preadapted sources along with those below 1000 m in the mountains to the South. When examining seed sources from across the entire species range, many stands are predicted to increase the adaptedness of all stands in the Santa Rosa Plateau, such as those near San Francisco, San Jose, Los Angeles, and San Diego to name a few (Figure S16). When examining the adaptedness of potential planting locations if stands in each focal site were moved across the range, all three focal sites had similar patterns of predicted low adaptedness when moved South or very far North, but relatively high adaptedness in the rest of the range (Figure S17).

## 4 Discussion

Based on the reduction in adaptedness between current spatial patterns of genetic gradients and those needed under predicted future climate, several stands of coast live oak across California are predicted to be more at risk of maladaptation in the future. At a finer scale, within TNC preserves that provide protection of their ecosystems and biodiversity, we also find variation in the suitability of local seed sources for future climate. We suggest that transferring seeds from stands identified as good seed sources for each focal site could enhance the adaptedness of stands more at risk of maladaptation to future climate. Coast live oak has very low genetic structure, similar to what is found in other California oak species (e.g., Craft and Ashley 2007; Ashley et al. 2018; Gugger et al. 2021; Mead et al. 2024; Buck et al. 2025, 2026). Despite the high geographic and ecological heterogeneity of the California landscape, apparently high gene flow and large effective population sizes result in very low genetic differentiation among regions based on data from primarily neutral loci across the whole genome sequences. The findings based on the ADMIXTURE analysis that all of our samples fall within one cluster is further evidence of the genetic connectedness across the species range. In contrast, our analysis of putative adaptive genetic variation reveals climate‐associated trends across the species range, indicating local adaptation despite high gene flow (Sork and Smouse 2006).

### 4.1 Adaptedness of Coast Live Oak Stands to Future Climate

Our landscape genomic analyses reveal that coast live oak's distribution represents a mosaic of stands with predicted high and low adaptedness across its range. Overall, lower adaptedness is predicted in the HadGEM2‐ES climate model than the CNRM‐CM5 model due to their differences in future climate regimes for California, where HadGEM2‐ES predicts a hotter and drier future than CNRM‐CM5 (Stocker et al. 2014). This trend, along with climate water deficit being one of the most important bioclimatic variables in explaining adaptive diversity (Figures S6–S8), implicates the importance of water availability in coast live oak systems, despite their drought tolerance (Chacon et al. 2020). Unsurprisingly, the rate of adaptedness decline from 2040–2069 to 2070–2099 is sharper in both models' RCP 8.5 scenario than in RCP 4.5, likely due to the continually increasing emissions modeled in 8.5 compared to the leveling off in 4.5 (Meinshausen et al. 2011). Stands in the northernmost and southernmost extents of coast live oak's range are identified as most at risk of maladaptation to future climate, having the lowest levels of adaptedness. Some lower elevation stands along the Central Coast are predicted to be most preadapted to future climate, having the highest levels of adaptedness. These heterogeneous patterns of predicted levels of maladaptation risk suggest different management strategies will be needed on a site‐by‐site basis and a “one size fits all” approach to coast live oak conservation will likely not work.

Five of the eight climate scenarios led to predictions that unprotected lands contain stands with higher adaptedness to future climate than protected lands, while the other three scenarios predicted the opposite. These findings indicate that in a majority of modeled future climate scenarios, unprotected lands will contain stands with higher adaptedness to future climate and should thus be targeted for protection, because unprotected lands could be at risk of extirpation from future land use change and incompatible management. We encourage considering additional criteria other than adaptedness when acquiring new lands for preservation (e.g., corridors between oak ecosystems and habitat for endangered species), but when choosing among multiple sites, selecting those with high predicted adaptedness is likely to result in the preservation of healthier future populations. The most preadapted stands in unprotected lands to focus on would be those in coastal areas South of Salinas and near San Luis Obispo (Figure S18), as long as those lands provide opportunities for conservation goals, such as retaining ecosystem services or improving habitat connectivity. We recommend that future land acquisitions consider management outcomes that address transitions and impacts of climate change (Barbour and Kueppers 2012) through ecological and genomic models.

Genomic data will not always yield the same patterns as ecological data (Buck et al. 2026). For example, Sousa et al. (2022) examined tree cover shifts during a severe drought within one of our focal sites (the Jack and Laura Dangermond Preserve), allowing for the indirect comparison of future adaptedness predictions with oak cover change predictions based on past dieback from 1982 to 2020. In general, stands with low adaptedness did not necessarily correspond with predicted canopy cover decrease. We predict that the northwest corner of the preserve will have some of the lowest adaptedness values in the 2040–2069 and 2070–2099 time periods, while Sousa et al. predicted little to no oak cover change in those areas by 2020 (Figure S19). Another contrast between the two studies is that we predict milder values of adaptedness along the eastern lip of the preserve, but they predict ~10% oak cover change by 2020. The reasons for these discrepancies aren't immediately apparent, but in addition to the timescales, the spatial resolution (30 m in Sousa et al. vs. 270 m here), and the use of additional variables (geology and soil data) by Sousa et al. differ. Despite the differences in methods and predictions, both studies have found that water availability is highly important in predicting oak response, suggesting that conservation efforts should consider restoring habitats that have high water availability and identify genotypes with higher drought tolerance. Future research could also incorporate soil data directly into genomic models and integrate ecological and genomic predictions to get a more accurate assessment of future oak cover change.

### 4.2 Managing Coast Live Oak Using TNC Preserves

The mosaic of predicted adaptedness throughout coast live oak's range suggests multiple management strategies are needed that match the respective stand's adaptedness and the goals of various management entities. For coast live oak stands that are predicted to be most preadapted, protection of existing trees through compatible land management practices (e.g., cattle grazing, prescribed fire, invasive plant control, and feral pig management) or restoration of previously removed oak habitat (e.g., after catastrophic fire or land clearing for development) should be the focus of land managers, ensuring that adequate recruitment of new seedlings is occurring. In contrast, stands predicted to be more at risk of maladaptation may require seed transfer within or between properties to retain oak ecosystem function because oak dispersal rates will likely not outpace rapid climate change (e.g., Sork et al. 2010) and long generation times limit the pace of rapid evolution (Malcolm et al. 2002; Aitken et al. 2008; Dauphin et al. 2021). However, managers should carefully consider the risks associated with seed transfer, such as disease spread, phenological mismatch, genetic load, outbreeding depression, lack of adaptation to local soil types and microbiomes, and genetic swamping of local genotypes (Todesco et al. 2016; Grummer et al. 2022). Fortunately, in oaks, outbreeding depression is not expected due to their lack of genetic structure (Frankham et al. 2011) and the benefits of transferring preadapted seeds to reduce climate maladaptation will likely result in an increase, not decrease, in fitness. To assess the success of a seed transfer program, land managers could monitor the growth and survival of young trees, ensuring that the intended fitness benefits will be achieved.

Our results provide unexpected insights into appropriate sources for range‐wide seed transfer. Notably, stands in the northern extent of coast live oak's range, including those inhabiting the mountains to the east of San Francisco and San Jose, were predicted to be good seed sources for the southern focal preserves, increasing their future adaptedness. This finding is surprising given the anticipated direction of climate change and the expectation that southern populations would already be adapted to hotter and drier conditions. We concede that long‐distance seed transfer might disrupt the local genetic composition of coast live oak populations, but we promote seed sourcing locally. We point out that preservation of coast live oak and the ecosystem they support may outweigh the loss of local genotypes. Here, we demonstrated that local stands within 50 km of focal sites contain seed sources that will increase the adaptedness of focal sites, likely minimizing the risks that may come with long‐distance seed transfer. By investigating preserves with differing future stand adaptedness, this study allowed us to evaluate contrasting conservation needs and to develop corresponding management strategies, which are applicable to other protected lands. These genomic findings are already being used by The Nature Conservancy to inform restoration and management of coast live oak across their conservation estate.

Coast live oak stands within the Jack and Laura Dangermond Preserve are predicted to be preadapted to future climate change scenarios, with relatively high adaptedness compared to the rest of its range. In most parts of the preserve, land managers likely do not need to invest resources into finding preadapted seed sources to increase adaptedness, but instead can focus on maintaining and growing existing populations by helping to encourage steady recruitment and growth of new seedlings. This goal can be accomplished through land use management practices, such as selective cattle grazing to prevent herbivory, invasive species removal to prevent resource competition, and hydrological protections to ensure water availability into the future (Tyler et al. 2006; López‐Sánchez et al. 2014; McLaughlin et al. 2017). Tree cover change can be monitored through remote sensing methods like in Sousa et al. (2022) to ensure management strategies are effective.

Coast live oaks at the Irvine Ranch Open Space are predicted to have relatively low adaptedness values, suggesting stands are not as preadapted to future climate scenarios. Coast live oak is the only upland tree within the Irvine Ranch Open Space, and the already low cover in this oak savanna ecosystem (only 4%) underscores the importance of ensuring existing populations do not decline further. Land managers can implement either low intensity or high intensity restoration strategies depending on their goals. If they aim to increase ecosystem services or return to a pre‐disturbance state of the site, high intensity restoration aimed at both increasing oak abundance and stand adaptedness can be implemented, especially if the site has a history of oak removal or destructive land use practices. To maintain current ecosystem services, land managers could employ low intensity restoration focused on increasing current stand adaptedness. In either strategy, preadapted seeds can be sourced from a few stands within the preserve, but the low stand presence limits the availability of resilient options. Local seed sources can still be found within 50 km in the Chino, Puente, and San Jose hills that will increase overall adaptedness and could be used to increase stand sizes.

The Santa Rosa Plateau stands of coast live oak are also predicted to be more at risk of maladaptation to future climate. Land managers could again focus on increasing adaptedness within the preserve, but with the higher oak coverage, they can more readily find preadapted seeds to use, especially in the southern extent of the preserve. If resources are limited, management efforts could focus on increasing adaptedness within the stands predicted to be most at risk of maladaptation to future climate, such as higher elevation stands, by sourcing seeds from the lower elevation stands. The preserve's combination of public open access and government‐owned land provides unique restoration opportunities. The public‐facing portion of the range would allow for community engagement in restoration efforts and monitoring, while ensuring balanced land use. The government‐owned portion would allow for the testing of restoration methods, with seedling caging to prevent rodent herbivory and deer browsing.

Ensuring the survival of seedlings through caging, managed grazing, and irrigation will be vital to restoration efforts, especially in preserves with limited resources that cannot afford multiple collecting and planting efforts. Raising coast live oak seeds in a nursery before outplanting could improve survival, likely due to the inherent protection and provided water, and result in larger seedlings than direct acorn plantings (Parikh and Gale 1998). However, direct planting is still a viable option, especially if acorns are protected from gophers and other herbivores (e.g., Tyler et al. 2008). Efforts should be taken to protect the seedlings, such as caging to prevent rodent access and deer grazing, which have been shown to significantly increase survival (almost 40% higher in Parikh and Gale 1998; also see Tyler et al. 2008). Land management strategies, such as non‐native annual grass removal through compatible grazing (e.g., a focus on winter season grazing versus summer, when non‐oak forage resources are more stressed and oaks are potentially more threatened by grazing and browsing) could also improve the survival of seedlings (Hall et al. 1992; Tyler et al. 2008).

By examining three TNC focal preserves, we were able to assess fine‐scale variation in the adaptedness of a foundation tree species and develop implementable, genomic‐informed conservation strategies broadly applicable to coast live oak and other foundation tree species. Predicted risk of climate maladaptation varied markedly even within individual preserves, consistent with evidence from other oak systems showing substantial individual‐level heterogeneity in tree response (Goetz et al. 2026). Preadapted seed sources were found locally within a preserve—or within 50 km in cases with low tree cover—reducing the need for state‐wide seed transfer and its associated risks (Grummer et al. 2022). However, for stands with extremely low predicted adaptedness, suitable seed sources may not be available and restoration may not be an effective use of management resources. Admittedly, integrating landscape genomics with common garden experiments provides a more robust assessment of maladaptation risk and seed sourcing (De Kort et al. 2014; Lepais and Bacles 2014; Aitken et al. 2024), but both sets of data are not always available, making landscape genomics a practical and informative first step.

### 4.3 Caveats to Our Study

Adaptedness (genomic offset as an alternative terminology) is a relatively new concept and, as such, its methods and the robustness of interpretation are still being evaluated (Capblancq et al. 2020; Fitzpatrick et al. 2021; Rellstab et al. 2021; Láruson et al. 2022; Lind et al. 2024; Lotterhos 2024; Lind and Lotterhos 2024). Adaptedness values are relative, meaning they can show stands that are predicted to be more or less at risk for maladaptation under future climates, but more research is needed to assess how the differences in adaptedness values translate into fitness differences. Additionally, adaptedness predictions can become less accurate when extrapolating to novel environments outside of the range of climate data used in training (Lind and Lotterhos 2024), and such novel environments are expected to increase with climate change (Lotterhos et al. 2021; Mahony et al. 2017). Future studies could examine the fitness consequences of genomic predictions by ground‐truthing adaptedness metrics with greenhouse or common garden data (Capblancq et al. 2020; Fitzpatrick et al. 2021; Rellstab et al. 2021; Láruson et al. 2022; Lind et al. 2024). One way this validation can be done is by raising multiple progeny from sequenced maternal sources in a common garden or at the restoration site and correlating the estimated relative fitness with the predicted genomic adaptedness (Lotterhos 2024). Genotypes with higher predicted adaptedness values in the common garden's climate are expected to have higher fitness in that environment, so a strong positive correlation would indicate high predictive performance of the adaptedness forecasts. Some studies have begun to tackle this issue, with most finding a correlation between offset and maladaptation (Fitzpatrick et al. 2021; Láruson et al. 2022; Lind and Lotterhos 2024). Similar studies are underway in oaks, where fitness data from a long‐term provenance study of valley oak is being compared to landscape genomic adaptedness predictions, and comparisons of seed sources for island oak and coast live oak restoration are being initiated by TNC on Santa Cruz Island, CA.

While utilizing landscape genomic statistical models is promising for predicting relative risk of maladaptation, method performance can vary depending on landscape types, strength of local adaptation, and marker sets (Lind and Lotterhos 2024). Here, multiple adaptedness methods are implemented and produced relatively similar predictions, but it is difficult to know which method has the most accurate predictions in areas of discordance. The slight discrepancies in patterns are likely due to how each model is built and their assumptions. For example, GDM and GF are nonlinear while RDA is linear, which means RDA patterns could be misleading in the presence of nonlinear genetic‐environmental relationships, a scenario likely in somewhere as geographically and ecologically complex as California. Additionally, GDM uses genetic distances while RDA and GF use allele frequencies, so GDM patterns could be influenced by uneven sampling schemes, a potential drawback of our study. A recent study by Bishop et al. (2025) simulated a large number of complex datasets to explore the effects of sampling design on landscape genomic analyses, finding that GEA analyses are relatively robust as long as environmental and geographic space are adequately covered. They were able to detect IBD and adaptive alleles with a minimum of 100 individuals—we have 171 here. However, we acknowledge more sampling (at least 200–400 individuals as Selmoni et al. 2020 suggest) would increase statistical power, potentially revealing even stronger patterns of local adaptation. The differences in how each method builds GEA relationships are elucidated in the importance of climate variables in each model, where GF ranks maximum temperature and climate water deficit as the two most important (Figure S6), GDM ranks climate water deficit then minimum temperature (Figure S7), and RDA ranks minimum temperature then soil recharge (Figure S8). In a recent comparison of offset methods, Archambeau et al. (2026) demonstrated that the predictions among methods varied substantially, with no single method consistently outperforming the others in predicting mortality rates. Given these factors, it is better to model adaptedness using multiple methods (Fitzpatrick and Keller 2015; Archambeau et al. 2026).

Another caveat to the use of GEAs is the challenge of controlling for genetic structure in species with low genetic structure and high geographic heterogeneity. Controlling for geography can reduce power and increase false positives in RDA outlier tests, especially in systems with low population structure (Forester et al. 2018; Lotterhos 2024; Bishop et al. 2025). Here, PC1 was strongly correlated with maximum temperature and climate water deficit (Table S4) so it is likely that accounting for PC1 in our pRDA removed some adaptive signal from those variables. However, the climate variables that explained the most variation in each GEA method (GF = maximum temperature and climate water deficit; RDA = minimum temperature and soil recharge; GDM = climate water deficit and minimum temperature) did not change between runs when accounting for PC1 and not (Figure S20); thus we are less concerned about this issue in the present study, but all studies should address the potential limitation.

In this study, we utilized an experimental design that uses landscape genomic predictions based on individual trees and predicts adaptedness at the resolution of our climate data (270 m × 270 m grid cells). An assumption of these models is that the grid cell contains a genotype locally adapted to the grid cell's current climate. It is possible that not all trees within that grid cell have equal levels of adaptedness to future climates, so until tree‐level climate data is made available, more work needs to assess this potential limitation. However, our analyses demonstrate that using grid cell‐resolution predictions will result in improvement of future populations.

Additionally, lag adaptation, where long‐lived species remain adapted to past climates and are consequently maladapted to current conditions, has been documented in California populations of valley oak (Browne et al. 2019; Goetz et al. 2026). While it has not been studied in coast live oak, lag adaptation would violate the assumptions of local adaptation in GEAs (Rellstab et al. 2016, 2021), but could be accounted for by using climate models from the period coast live oak is adapted to. If present, the risks of maladaptation observed here would likely be exacerbated (Browne et al. 2019; Goetz et al. 2026). Nonetheless, our conservation recommendations of using genomic‐informed seed sourcing would likely not change because the use of climate‐adapted seed transfer is still likely to mitigate future maladaptation from climate change (Goetz et al. 2026).

Finally, the models employed here associated abiotic data (bioclimatic and geographic variables) with genomic variation to find patterns of local adaptation and make adaptedness predictions; however, we acknowledge that this focus does not fully capture all interactions and threats to coast live oak. Oak management and restoration will need to consider other factors such as competition, habitat connectivity, native herbivores, cattle grazing patterns, and even disease resistance. Future research that develops methods to incorporate these factors could give further insight into which threats specific populations are most at risk for and what actions managers can take to mediate them.

## 5 Conclusions

The species range of coast live oak is comprised of a mosaic of adaptedness: stands in the north and south seem more at risk for maladaptation to future climate, while those along the central coast of California seem to be less at risk. A large portion (40%) of coast live oak's range is currently under some form of protected status, but those lands may be jeopardized in the future by warmer temperatures predicted by several climate scenarios. Those protected stands predicted to be more at risk of maladaptation to future climate and the ecosystems they support would benefit from active genomic‐informed management. In contrast, currently unprotected lands with coast live oak stands predicted to be more preadapted to future climate are great candidates for acquisition. Some coast live oak stands, even those with higher risk of maladaptation, could be important for preservation for other reasons, such as being corridors of connectivity or habitat for endangered species. Of the three focal TNC preserves examined here, two contain stands of low adaptedness, with one having coast live oak as its only upland tree. If restoration projects are undertaken, managers could use this genomic information to select seed source trees and then track and monitor which trees perform best to improve future management efforts. This study illustrates how collaboration between academic researchers and conservation professionals can inform management of a foundational tree species, offering a model for state, federal, and non‐profit agencies.

## Funding

Funding for this study was provided by The Nature Conservancy, California chapter.

## Conflicts of Interest

The authors declare no conflicts of interest.

## Supporting information


**Data S1:** eva70224‐sup‐0001‐supinfo.docx.
**Figure S1:** The Nature Conservancy's conservation estate across California. Each circle represents a managed property, with the size of the circle corresponding to the size of the preserve.
**Figure S2:** Environmental gradients captured by our sampling design (blue) versus the entire species distribution of coast live oak (red).
**Figure S3:** Graphical illustration of how adaptedness after seed transfer is calculated. (A) To find potential seed sources that would be preadapted to a future planting site, reverse adaptedness is calculated between the adaptive variation needed in the future climate (T2) of a recipient cell and the adaptive variation currently present (T1) in potential donor cells. Adaptedness values can then be mapped to show stands' adaptedness if transferred to a given planting site. (B) To find potential future planting sites that current seed sources would be preadapted to, forward adaptedness is calculated between the adaptive variation currently present (T1) in donor cells and the adaptive variation needed in the future climate (T2) of a potential recipient cells. Adaptedness values can then be mapped to show stands' adaptedness if given seeds were transferred into the site.
**Figure S4:** (A) Isolation by distance results showing geographic distance in kilometers and genetic distance (calculated as the Euclidean distance between individual genotypes) with each dot representing a pairwise comparison between individuals. The trend was significant (*p* = 0.001), suggesting that geographic distance is positively correlated with genetic distance. (B) Isolation by environment accounting for geography results showing environmental distance (calculated as the Euclidean distances of scaled mean environmental variables at each individual's location) and genetic distance (calculated as the Euclidean distance between individual genotypes) with each dot representing a pairwise comparison between individuals. The trend was not significant (*p* = 0.53), suggesting that environmental distance is not significantly correlated with genetic distance.
**Figure S5:** Genomic turnover showing adaptive composition interpolated across coast live oak's range by gradient forest models. Larger differences in colors indicate larger differences in adaptive composition. Overlaid boxes contain enlarged adaptive composition of the three Nature Conservancy (TNC) focal sites.
**Figure S6:** Importance of bioclimatic variables in the gradient forest models.
The left graph shows the unweighted average of split importances across loci, or how often a predictor variable was used to split regression trees, while the right graph shows the split importance weighted by the variance explained for each locus. SNPs with non‐zero *R* ^2^ in the GF model (942 SNPs) had predictive power and were thus used by the model in the turnover and offset predictions.
**Figure S7:** Importance of bioclimatic variables in the generalized dissimilarity models, with each as a function of variation explained. Climate water deficit cwd is the most important predictor of genetic dissimilarity, followed by minimum temperature (tmn), soil recharge (rch), maximum temperature (tmax), and soil storage (str). The first graph includes environmental distance by observed genetic distance, with the positive slope indicating isolation by environment, which is expected when examining genotype‐environment associations. Note that runoff is not pictured because the model did not find it explained a significant amount of variation.
**Figure S8:** Distribution of genomic variation in RDA space using all loci, with RDA1 accounting for 17.8% of variation and RDA2 accounting for 17.1%. The 1741 outlier loci above the Bonferroni‐corrected significance threshold (*α* = 0.01/*n*) are colored orange, while the other 946,645 loci are colored grey. The strength of each bioclimatic variable on variation is represented by the black loading arrows, with longer arrows representing more importance in explaining variation in the direction they are pointing.
**Figure S9:** Adaptedness predictions using gradient forest for RCP 4.5 and 8.5, climate models HadGEM2‐ES and CNRM‐CM5, and 30‐year average time periods 2040–2069 and 2070–2099. Lighter colors indicate higher adaptedness values.
**Figure S10:** Adaptedness predictions using redundancy analysis (RDA) for RCP 4.5 and 8.5, climate models HadGEM2‐ES and CNRM‐CM5, and 30‐year average time periods 2040–2069 and 2070–2099. Lighter colors indicate higher adaptedness values.
**Figure S11:** Adaptedness predictions using generalized dissimilarity models (GDM) for RCP 4.5 and 8.5, climate models HadGEM2‐ES and CNRM‐CM5, and 30‐year average time periods 2040–2069 and 2070–2099. Lighter colors indicate higher adaptedness values.
**Figure S12:** Adaptedness predictions of the three methods (gradient forest, GF; generalized dissimilarity models, GDM; redundancy analysis, RDA) for HadGEM2‐ES RCP 8.52070–2099. Lighter colors indicate higher adaptedness values. Adaptedness values are not directly comparable among methods, but comparisons of patterns of highest and lowest adaptedness are Valid.
**Figure S13:** Procrustes residuals from pairwise adaptedness method comparison (GF, gradient forest, GDM, generalized dissimilarity model; RDA, redundancy analysis) for HadGEM2‐ES RCP 8.52070‐2099. Darker colors indicate larger differences between methods. Values are not directly comparable among analyses but comparisons of patterns of largest and smallest differences are valid.
**Figure S14:** (A) Adaptedness values of donor cells if transferred from the entire coast live oak range to the Jack and Laura Dangermond Preserve modeled in HadGEM‐ES2 RCP 8.52070–2099, with lighter colors indicating higher average adaptedness of the Jack and Laura Dangermond Preserve if the grid cell was used as a seed source. (B) Seed source priority from the entire coast live oak range to the Jack and Laura Dangermond Preserve modeled in HadGEM‐ES2 RCP 8.52070–2099, with greener colors indicating a higher percentage of the Jack and Laura Dangermond Preserve that would have increased adaptedness if the grid cell was used as a seed source.
**Figure S15:** (A) Adaptedness values of donor cells if transferred from the entire coast live oak range to the Irvine Ranch Open Space modeled in HadGEM‐ES2 RCP 8.52070–2099, with lighter colors indicating higher average adaptedness of the Irvine Ranch Open Space if the grid cell was used as a seed source. (B) Seed source priority from the entire coast live oak range to the Irvine Ranch Open Space modeled in HadGEM‐ES2 RCP 8.52070–2099, with greener colors indicating a higher percentage of the Irvine Ranch Open Space that would have increased adaptedness if the grid cell was used as a seed source.
**Figure S16:** (A) Adaptedness values of donor cells from the entire coast live oak range to Santa Rose Plateau modeled in HadGEM‐ES2 RCP 8.52070–2099, with lighter colors indicating higher average adaptedness of the Santa Rosa Plateau if the grid cell was used as a seed source. (B) Seed source priority from the entire coast live oak range to the Santa Rosa Plateau modeled in HadGEM‐ES2 RCP 8.52070–2099, with greener colors indicating a higher percentage of the Santa Rosa Plateau that would have increased adaptedness if the grid cell was used as a seed source.
**Figure S17:** Forward adaptedness values from each focal site (A = Jack and Laura Dangermond Preserve, B = Irvine Ranch Open Space, C = Santa Rosa Plateau) to the entire coast live oak range modeled in HadGEM‐ES2 RCP 8.52070‐2099, with lighter colors indicating higher average adaptedness of each focal site if the grid cell was used as a planting location.
**Figure S18:** Adaptedness predictions within unprotected lands for climate model CNRM‐CM5, RCP 8.5, 2070–2099. Lighter colors indicate higher adaptedness values and thus less predicted maladaptation to future climate.
**Figure S19:** Side‐by‐side visual comparison of adaptedness results (A) within the Jack and Laura Dangermond Preserve modeled in this study, with the land classes (B) and corresponding oak cover change (C) predicted by Sousa et al. (2022). It is important to note the scale of adaptedness values in A are relative to the Jack and Laura Dangermond Preserve, not to the entire coast live oak range as shown in Figure 3. This scale was selected to emphasize the differences in adaptedness values within the preserve.
**Figure S20:** Importance of climate variables in explaining putatively adaptive variation when including PC1 (A–C) and not (D–F) in the pRDA to find outlier loci. A and D show generalized dissimilarity model (GDM) variable importance, B and E show gradient forest (GF) variable importance, and C and F show redundancy analysis (RDA) variable importance.
**Table S1:** Ten bioclimatic variables used in genotype‐environment associations.
**Table S2:** Partial redundancy analysis results.
**Table S3:** Permutation tests examining significance of each RDA axes in the constrained pRDA using a subset of 100,000 loci and 250 permutations.
**Table S4:** Correlation matrix of genetic structure vs geography. Genetic structure is represented by PC axes and geography is represented by latitude, longitude, and latitude × longitude.
**Table S5:** Gene ontology of climate‐associated SNPs within 1kbp of a known gene.
**Table S6:** Results of ANOVA of protection status, climate model, RCP, and year on adaptedness.
**Table S7:** Procrustes spatial correlations among adaptedness methods for each climate model, RCP, and year. All correlations were significant (*p* < 0.001).
**Table S8:** Spearman rank correlations (*ρ*) among adaptedness methods for each climate model, RCP, and year.
**Table S9:** Mean and standard error of adaptedness values for each Model, RCP, year, and protection status shown in Figure 4.
**Table S10:** Tukey's posthoc test of differences among factors in the nested ANOVA (Adaptedness ~ Protection * Model/RCP/Year) examining adaptedness in unprotected vs protected lands.

## Data Availability

Raw sequencing reads and metadata are available at NCBI under BioProject PRJNA1433066 (https://www.ncbi.nlm.nih.gov/bioproject/1433066). Locality data for collected samples are available as Table 1 and in the BioProject metadata. Analysis scripts are available at Github (https://github.com/ryancollinbuck/qagr) and archived at Dryad (https://datadryad.org/dataset/10.5061/dryad.rv15dv4p4).
