# Supplementary material for: Landscape Genomic Analyses of Quercus agrifolia Née Predict Patterns of Adaptedness to Future Climate and Provide Guidance for Conservation
Source: Evol Appl. 2026 Apr 21;19(4):e70224. doi: 10.1111/eva.70224 (PMC13097008; doi:10.1111/eva.70224)
Supplement: Supplementary file 1 — Data S1: eva70224‐sup‐0001‐supinfo.docx. Figure S1: The Nature Conservancy's conservation estate across California. Each circle represents a managed property, with the size of the circle corresponding to the size of the preserve. Figure S2: Environmental gradients captured by our sampling design (blue) versus the entire species distribution of coast live oak (red). Figure S3: Graphical illustration of how adaptedness after seed transfer is calculated. (A) To find potential seed sources that would be preadapted to a future planting site, reverse adaptedness is calculated between the adaptive variation needed in the future climate (T2) of a recipient cell and the adaptive variation currently present (T1) in potential donor cells. Adaptedness values can then be mapped to show stands' adaptedness if transferred to a given planting site. (B) To find potential future planting sites that current seed sources would be preadapted to, forward adaptedness is calculated between the adaptive variation currently present (T1) in donor cells and the adaptive variation needed in the future climate (T2) of a potential recipient cells. Adaptedness values can then be mapped to show stands' adaptedness if given seeds were transferred into the site. Figure S4: (A) Isolation by distance results showing geographic distance in kilometers and genetic distance (calculated as the Euclidean distance between individual genotypes) with each dot representing a pairwise comparison between individuals. The trend was significant (p = 0.001), suggesting that geographic distance is positively correlated with genetic distance. (B) Isolation by environment accounting for geography results showing environmental distance (calculated as the Euclidean distances of scaled mean environmental variables at each individual's location) and genetic distance (calculated as the Euclidean distance between individual genotypes) with each dot representing a pairwise comparison between individuals. The trend was not sign [file EVA-19-e70224-s001.zip › eva70224-sup-0001-supinfo.docx]

Supplementary Materials for

**Landscape Genomic Analyses of *Quercus agrifolia* Née Predict Patterns of Adaptedness to Future Climate and Provide Guidance for Conservation**

R.C. Buck, H. S. Butterfield, E. Hiroyasu, J. Howard, J. Knapp, Z. Principe, and V. L. Sork

**Supplementary Methods**

*GF adaptedness* – Gradient Forest is a machine learning random forest technique that models multivariate allele frequencies as ensembled nonlinear regression functions of environmental variables (Ellis et al., 2012; Fitzpatrick and Keller, 2015). Future climate variables are then transformed using fitted forest functions to model predicted future adaptive genetic composition. Offset is calculated as the multivariate distance between the predicted future and current adaptive genetic compositions. Adaptedness is calculated by multiplying offset by -1 so that stands that are more preadapted to future climate have larger adaptedness values. The ‘gradientForest’ function was run on the 1,741 pRDA-identified putatively adaptive loci with the number of trees set to 500, correlation threshold to 0.5, and max level set to log_2_(0.368 × number of samples/2) (Ellis et al., 2012). Adaptive genomic turnover was then predicted for the whole range using the ‘predict’ function from the *gradientForest* R package and mapped using Fitzpatrick and Keller’s (2015) function ‘pcaToRaster’. Local genomic offset between the current and future adaptive variation at each climate model, RCP, and time period were calculated using Fitzpatrick and Keller’s (2015) code. Offset values were then transformed into adaptedness values by multiplying by -1, so that low offset values are now high on the adaptedness scale. The resulting adaptedness values were then mapped in ArcGIS^®^ Pro (ESRI).

*RDA adaptedness* – Redundancy analysis is a constrained ordination technique that models multivariate allele frequencies as a linear function of environmental variables to explain adaptive genetic variation (Capblancq and Forester, 2021). After fitting the RDA model with current climate variables, future predicted climate data are then projected into RDA space to predict the adaptive composition needed in future climates. The Euclidean distance between the predicted future and current adaptive genetic compositions in RDA space is calculated as offset. Adaptedness is calculated in the same way as above, by multiplying offset values by -1. Following Capblancq and Forester’s (2021) code, an adaptively enriched constrained RDA was modeled using the 1,741 pRDA-identified putatively adaptive loci, six noncollinear climate variables, and conditioned on the same geographical (latitude, longitude, and latitude x longitude) and population structure (PC1) variables as the original pRDA, keeping the same two RDA axes. Adaptive genetic turnover was then predicted for the whole range using Capblancq and Forester’s (2021) ‘adaptive_index’ function. Local genomic offset examines the genotype-environment discordance between a future climate cell and a current climate cell in the same location to evaluate the level of that stand’s risk of climate maladaptation (Gougherty et al., 2021). Local genomic offset between the current and future adaptive variation at each climate model, RCP, and time period were calculated using Capblancq and Forester’s (2021) ‘genomic_offset’ function keeping two RDA axes as in the original pRDA. Offset values were then transformed into adaptedness values by multiplying by -1, so that low offset values are now high on the adaptedness scale. The resulting adaptedness values were then mapped in ArcGIS^®^ Pro (ESRI, 2025).

*GDM adaptedness* – Generalized dissimilarity modeling is a nonlinear regression technique that fits pairwise genetic distances between individuals to pairwise environmental distances using I-spline transformations (Ferrier et al., 2007; Fitzpatrick and Keller, 2015). Once the GDM is fitted with current climate variables, future predicted climate data are then transformed by the I-spline to predict the adaptive composition needed in future climates. Offset is calculated as the Euclidean distance between the predicted future and current adaptive variations. Adaptedness is calculated in the same way as above, by multiplying offset values by -1. Adapting Fitzpatrick and Keller’s (2015) code, the genetic distance between the 1,741 pRDA-identified putatively adaptive loci was calculated using the Bray method of the ‘vegdist’ function in the R package *vegan*. A GDM then fit the genetic distances as a function of environmental distances using the ‘gdm’ function in the *gdm* package (Fitzpatrick et al., 2024). Adaptive genetic turnover was then predicted for the whole range using the ’gdm.transform’ function and mapped using Fitzpatrick and Keller’s (2015) function ‘pcaToRaster’. Local genomic offset between the current and future adaptive variation at each climate model, RCP, and time period were calculated using Fitzpatrick and Keller’s (2015) code. Offset values were then transformed into adaptedness values by multiplying by -1, so that low offset values are now high on the adaptedness scale. The resulting adaptedness values were then mapped in ArcGIS^®^ Pro (ESRI).

**Supplementary Table 1.** Ten bioclimatic variables^1^ used in genotype-environment associations.

| **Variable abbreviation** | **Variable full name** | **Units** | **Variable type** | **Description (from U.S. Geological Survey)** |
| --- | --- | --- | --- | --- |
| aet | Actual evapotranspiration | mm | Hydrologic | Amount of water that evaporates from the surface and is transpired by plants, summed annually |
| cwd | Climate water deficit | mm | Hydrologic | Annual evaporative demand that exceeds available water, summed annually |
| pck | Snowpack | mm | Hydrologic | Amount of snow as a water equivalent that is accumulated per month, summed annually |
| pet | Potential evapotranspiration | mm | Climate | Amount of water that can evaporate from the ground surface or be transpired by plants, summed annually |
| ppt | Precipitation | mm | Climate | Total monthly precipitation (rain or snow), summed annually |
| rch | Recharge | mm | Hydrologic | Amount of water that penetrates below the root zone, summed annually |
| run | Runoff | mm | Hydrologic | Amount of water that becomes stream flow, summed annually |
| str | Soil water storage | mm | Hydrologic | Amount of water stored in the soil annually |
| tmn | Minimum air temperature | °C | Climate | Minimum monthly temperature, averaged annually |
| tmx | Maximum air temperature | °C | Climate | Maximum monthly temperature, averaged annually |

^1^Variables are 30-year summaries extracted from the Basin Characterization Model at 270m resolution (Stern et al., 2024)

**Supplementary Table 2.** Partial redundancy analysis^1^ results.

| **pRDA model** | **Inertia** | **R^2^** | **Pr(>F)** | **Proportion of explainable variance** | **Proportion of total variance** |
| --- | --- | --- | --- | --- | --- |
| Full : W ~ clim + geo + pop | 7671 | 0.0146 | 0.001 | 1 | 0.073 |
| pure Climate : W ~ clim \| (geo + pop) | 3811 | 0.0013 | 0.001 | 0.497 | 0.036 |
| pure Geography : W ~ geo \| (clim + pop) | 2179 | 0.0034 | 0.001 | 0.284 | 0.021 |
| pure Structure : W ~ pop \| (clim + geo) | 1088 | 0.0047 | 0.001 | 0.142 | 0.010 |
| Confounded (clim / geo / pop) | 593 |  |  | 0.077 | 0.006 |
| Total unexplained | 98060 |  |  |  | 0.927 |
| Total inertia | 105700 |  |  |  | 1 |

^1^Redundancy analyses tested the effects of climate, geography, population structure, and all three on genetic variation (W = genomic data, clim = six uncorrelated climate variables; geo = latitude, longitude, and latitude x longitude; pop = PC1).

**Supplementary Table 3.** Permutation tests examining significance of each RDA axes in the constrained pRDA using a subset of 100,000 loci and 250 permutations.

|  | **Df** | **Variance** | **F** | **Pr(>F)** |
| --- | --- | --- | --- | --- |
| RDA1 | 1 | 71.8 | 1.108 | 0.004 |
| RDA2 | 1 | 70.6 | 1.090 | 0.004 |
| RDA3 | 1 | 67.4 | 1.041 | 0.645 |
| RDA4 | 1 | 65.6 | 1.013 | 0.952 |
| RDA5 | 1 | 64.3 | 0.992 | 0.972 |
| RDA6 | 1 | 62.8 | 0.971 | 0.865 |

**Supplementary Table 4.** Correlation matrix of genetic structure vs geography. Genetic structure is represented by PC axes and geography is represented by latitude, longitude, and latitude × longitude.

|  | **Latitude** | **Longitude** | **Latitude × Longitude** |
| --- | --- | --- | --- |
| **PC1** | -0.92 | 0.80 | -0.86 |
| **PC2** | 0.01 | -0.51 | 0.37 |
| **PC3** | -0.31 | 0.21 | -0.25 |
| **PC4** | 0.05 | -0.01 | 0.02 |

**Supplementary Table 6.** Results of ANOVA^1^ of protection status, climate model, RCP, and year on adaptedness.

|  | **Df** | **Sum sq** | **Mean Sq** | **F value** | **Pr(>F)** |
| --- | --- | --- | --- | --- | --- |
| Protection | 1 | 0.001 | 0.0008 | 367.6 | <1×10^-15^ |
| Model | 1 | 1.774 | 1.7741 | 816859.5 | <1×10^-15^ |
| Protection:Model | 1 | 0.009 | 0.0088 | 4067.5 | <1×10^-15^ |
| Protection:Model:RCP | 4 | 4.633 | 1.1582 | 533294 | <1×10^-15^ |
| Protection:Model:RCP:Year | 8 | 5.621 | 0.7026 | 32510.8 | <1×10^-15^ |
| Residuals | 1847912 | 4.013 | <1×10^-5^ |  |  |

^1^(Adaptedness ~ Protection * Model/RCP/Year) treating year (2040-2069 or 2070-2099) as nested within RCP (4.5 or 8.5) which is nested within climate model (CNRM-CM5 or HadGEM2-ES). All interactions were significant.

**Supplementary Table 7.** Procrustes spatial correlations among adaptedness methods for each climate model, RCP, and year. All correlations were significant (p < 0.001).

| **Model** | **GDM vs GF** | **GDM vs RDA** | **GF vs RDA** |
| --- | --- | --- | --- |
| H_85_2070 | 0.1346 | 0.3916 | 0.4064 |
| H_85_2040 | 0.1472 | 0.2675 | 0.7249 |
| H_45_2070 | 0.0421 | 0.2576 | 0.5563 |
| H_45_2040 | 0.1301 | 0.2153 | 0.5649 |
| C_85_2070 | 0.2507 | 0.3521 | 0.7357 |
| C_85_2040 | 0.0893 | 0.1319 | 0.4051 |
| C_45_2070 | 0.0256 | 0.0439 | 0.3635 |
| C_45_2040 | 0.1320 | 0.0581 | 0.2372 |
| **Average** | **0.1190** | **0.2148** | **0.4993** |

**Supplementary Table 8.** Spearman rank correlations (ρ) among adaptedness methods for each climate model, RCP, and year.

| **Model** | **GDM vs GF** | **GDM vs RDA** | **GF vs RDA** |
| --- | --- | --- | --- |
| H_85_2070 | 0.0796 | 0.3621 | 0.4641 |
| H_85_2040 | 0.1489 | 0.3159 | 0.6972 |
| H_45_2070 | 0.0367 | 0.2913 | 0.4962 |
| H_45_2040 | 0.1139 | 0.1887 | 0.5291 |
| C_85_2070 | 0.3113 | 0.3869 | 0.6608 |
| C_85_2040 | 0.1117 | 0.1721 | 0.2941 |
| C_45_2070 | 0.0644 | 0.0470 | 0.2749 |
| C_45_2040 | 0.1592 | 0.0664 | 0.2475 |
| **Average** | **0.1282** | **0.2288** | **0.4580** |

**Supplementary Table 9.** Mean and standard error of adaptedness values for each Model, RCP, year, and protection status shown in Fig. 4

| **Model** | **RCP** | **Year** | **Protection** | **mean_adaptedness** | **se_adaptedness** |
| --- | --- | --- | --- | --- | --- |
| C | 45 | 2040 | Protected | -0.00522 | 2.65E-06 |
| C | 45 | 2040 | Unprotected | -0.00498 | 2.79E-06 |
| C | 45 | 2070 | Protected | -0.00708 | 3.53E-06 |
| C | 45 | 2070 | Unprotected | -0.00693 | 3.61E-06 |
| C | 85 | 2040 | Protected | -0.00669 | 2.83E-06 |
| C | 85 | 2040 | Unprotected | -0.0066 | 2.84E-06 |
| C | 85 | 2070 | Protected | -0.0111 | 3.68E-06 |
| C | 85 | 2070 | Unprotected | -0.0109 | 3.64E-06 |
| H | 45 | 2040 | Protected | -0.0065 | 3.16E-06 |
| H | 45 | 2040 | Unprotected | -0.00661 | 2.74E-06 |
| H | 45 | 2070 | Protected | -0.0086 | 3.09E-06 |
| H | 45 | 2070 | Unprotected | -0.00878 | 2.47E-06 |
| H | 85 | 2040 | Protected | -0.00873 | 3.67E-06 |
| H | 85 | 2040 | Unprotected | -0.00891 | 2.87E-06 |
| H | 85 | 2070 | Protected | -0.0135 | 2.8E-06 |
| H | 85 | 2070 | Unprotected | -0.0134 | 2.33E-06 |

**Supplementary Table 10.** Tukey’s posthoc test of differences among factors in the nested ANOVA (Adaptedness ~ Protection * Model/RCP/Year) examining adaptedness in unprotected vs protected lands.

|  | **diff** | **lwr** | **upr** | **p adj** |
| --- | --- | --- | --- | --- |
| Unprotected-Protected | -4.16E-05 | -4.59E-05 | -3.74E-05 | <1.00E-15 |
| H-C | 0.00196 | 0.001955 | 0.001964 | <1.00E-15 |
| Unprotected:C-Protected:C | -1.80E-04 | -1.88E-04 | -0.00017 | <1.00E-15 |
| Protected:H-Protected:C | 1.81E-03 | 1.81E-03 | 0.001823 | <1.00E-15 |
| Unprotected:H-Protected:C | 1.91E-03 | 1.90E-03 | 0.00192 | <1.00E-15 |
| Protected:H-Unprotected:C | 1.99E-03 | 1.99E-03 | 0.002003 | <1.00E-15 |
| Unprotected:H-Unprotected:C | 2.09E-03 | 2.08E-03 | 0.0021 | <1.00E-15 |
| Unprotected:H-Protected:H | 9.68E-05 | 8.89E-05 | 0.000105 | <1.00E-15 |
| Unprotected:C:45-Protected:C:45 | -1.94E-04 | -2.07E-04 | -1.81E-04 | <1.00E-15 |
| Protected:H:45-Protected:C:45 | 1.40E-03 | 1.38E-03 | 1.41E-03 | <1.00E-15 |
| Unprotected:H:45-Protected:C:45 | 1.54E-03 | 1.53E-03 | 1.55E-03 | <1.00E-15 |
| Protected:C:85-Protected:C:45 | 2.74E-03 | 2.73E-03 | 2.76E-03 | <1.00E-15 |
| Unprotected:C:85-Protected:C:45 | 2.58E-03 | 2.56E-03 | 2.59E-03 | <1.00E-15 |
| Protected:H:85-Protected:C:45 | 4.97E-03 | 4.96E-03 | 4.99E-03 | <1.00E-15 |
| Unprotected:H:85-Protected:C:45 | 5.03E-03 | 5.01E-03 | 5.04E-03 | <1.00E-15 |
| Protected:H:45-Unprotected:C:45 | 1.59E-03 | 1.58E-03 | 1.61E-03 | <1.00E-15 |
| Unprotected:H:45-Unprotected:C:45 | 1.74E-03 | 1.72E-03 | 1.75E-03 | <1.00E-15 |
| Protected:C:85-Unprotected:C:45 | 2.94E-03 | 2.92E-03 | 2.95E-03 | <1.00E-15 |
| Unprotected:C:85-Unprotected:C:45 | 2.77E-03 | 2.76E-03 | 2.78E-03 | <1.00E-15 |
| Protected:H:85-Unprotected:C:45 | 5.17E-03 | 5.16E-03 | 5.18E-03 | <1.00E-15 |
| Unprotected:H:85-Unprotected:C:45 | 5.22E-03 | 5.21E-03 | 5.23E-03 | <1.00E-15 |
| Unprotected:H:45-Protected:H:45 | 1.43E-04 | 1.30E-04 | 1.56E-04 | <1.00E-15 |
| Protected:C:85-Protected:H:45 | 1.35E-03 | 1.33E-03 | 1.36E-03 | <1.00E-15 |
| Unprotected:C:85-Protected:H:45 | 1.18E-03 | 1.17E-03 | 1.19E-03 | <1.00E-15 |
| Protected:H:85-Protected:H:45 | 3.58E-03 | 3.56E-03 | 3.59E-03 | <1.00E-15 |
| Unprotected:H:85-Protected:H:45 | 3.63E-03 | 3.61E-03 | 3.64E-03 | <1.00E-15 |
| Protected:C:85-Unprotected:H:45 | 1.20E-03 | 1.19E-03 | 1.22E-03 | <1.00E-15 |
| Unprotected:C:85-Unprotected:H:45 | 1.04E-03 | 1.02E-03 | 1.05E-03 | <1.00E-15 |
| Protected:H:85-Unprotected:H:45 | 3.43E-03 | 3.42E-03 | 3.45E-03 | <1.00E-15 |
| Unprotected:H:85-Unprotected:H:45 | 3.48E-03 | 3.47E-03 | 3.50E-03 | <1.00E-15 |
| Unprotected:C:85-Protected:C:85 | -1.66E-04 | -1.79E-04 | -1.53E-04 | <1.00E-15 |
| Protected:H:85-Protected:C:85 | 2.23E-03 | 2.22E-03 | 2.25E-03 | <1.00E-15 |
| Unprotected:H:85-Protected:C:85 | 2.28E-03 | 2.27E-03 | 2.30E-03 | <1.00E-15 |
| Protected:H:85-Unprotected:C:85 | 2.40E-03 | 2.38E-03 | 2.41E-03 | <1.00E-15 |
| Unprotected:H:85-Unprotected:C:85 | 2.45E-03 | 2.44E-03 | 2.46E-03 | <1.00E-15 |
| Unprotected:H:85-Protected:H:85 | 5.06E-05 | 3.75E-05 | 6.38E-05 | <1.00E-15 |
| Unprotected:C:45:2040-Protected:C:45:2040 | -2.39E-04 | -2.60E-04 | -2.18E-04 | <1.00E-15 |
| Protected:H:45:2040-Protected:C:45:2040 | 1.28E-03 | 1.26E-03 | 1.30E-03 | <1.00E-15 |
| Unprotected:H:45:2040-Protected:C:45:2040 | 1.39E-03 | 1.36E-03 | 1.41E-03 | <1.00E-15 |
| Protected:C:85:2040-Protected:C:45:2040 | 1.47E-03 | 1.45E-03 | 1.50E-03 | <1.00E-15 |
| Unprotected:C:85:2040-Protected:C:45:2040 | 1.38E-03 | 1.36E-03 | 1.40E-03 | <1.00E-15 |
| Protected:H:85:2040-Protected:C:45:2040 | 3.51E-03 | 3.49E-03 | 3.53E-03 | <1.00E-15 |
| Unprotected:H:85:2040-Protected:C:45:2040 | 3.69E-03 | 3.67E-03 | 3.71E-03 | <1.00E-15 |
| Protected:C:45:2070-Protected:C:45:2040 | 1.86E-03 | 1.84E-03 | 1.88E-03 | <1.00E-15 |
| Unprotected:C:45:2070-Protected:C:45:2040 | 1.72E-03 | 1.69E-03 | 1.74E-03 | <1.00E-15 |
| Protected:H:45:2070-Protected:C:45:2040 | 3.38E-03 | 3.36E-03 | 3.40E-03 | <1.00E-15 |
| Unprotected:H:45:2070-Protected:C:45:2040 | 3.56E-03 | 3.54E-03 | 3.58E-03 | <1.00E-15 |
| Protected:C:85:2070-Protected:C:45:2040 | 5.88E-03 | 5.85E-03 | 5.90E-03 | <1.00E-15 |
| Unprotected:C:85:2070-Protected:C:45:2040 | 5.64E-03 | 5.62E-03 | 5.66E-03 | <1.00E-15 |
| Protected:H:85:2070-Protected:C:45:2040 | 8.30E-03 | 8.28E-03 | 8.32E-03 | <1.00E-15 |
| Unprotected:H:85:2070-Protected:C:45:2040 | 8.23E-03 | 8.20E-03 | 8.25E-03 | <1.00E-15 |
| Protected:H:45:2040-Unprotected:C:45:2040 | 1.52E-03 | 1.50E-03 | 1.54E-03 | <1.00E-15 |
| Unprotected:H:45:2040-Unprotected:C:45:2040 | 1.62E-03 | 1.60E-03 | 1.65E-03 | <1.00E-15 |
| Protected:C:85:2040-Unprotected:C:45:2040 | 1.71E-03 | 1.69E-03 | 1.74E-03 | <1.00E-15 |
| Unprotected:C:85:2040-Unprotected:C:45:2040 | 1.62E-03 | 1.60E-03 | 1.64E-03 | <1.00E-15 |
| Protected:H:85:2040-Unprotected:C:45:2040 | 3.75E-03 | 3.73E-03 | 3.77E-03 | <1.00E-15 |
| Unprotected:H:85:2040-Unprotected:C:45:2040 | 3.93E-03 | 3.91E-03 | 3.95E-03 | <1.00E-15 |
| Protected:C:45:2070-Unprotected:C:45:2040 | 2.10E-03 | 2.08E-03 | 2.12E-03 | <1.00E-15 |
| Unprotected:C:45:2070-Unprotected:C:45:2040 | 1.95E-03 | 1.93E-03 | 1.97E-03 | <1.00E-15 |
| Protected:H:45:2070-Unprotected:C:45:2040 | 3.62E-03 | 3.60E-03 | 3.64E-03 | <1.00E-15 |
| Unprotected:H:45:2070-Unprotected:C:45:2040 | 3.80E-03 | 3.78E-03 | 3.82E-03 | <1.00E-15 |
| Protected:C:85:2070-Unprotected:C:45:2040 | 6.11E-03 | 6.09E-03 | 6.14E-03 | <1.00E-15 |
| Unprotected:C:85:2070-Unprotected:C:45:2040 | 5.88E-03 | 5.86E-03 | 5.90E-03 | <1.00E-15 |
| Protected:H:85:2070-Unprotected:C:45:2040 | 8.54E-03 | 8.52E-03 | 8.56E-03 | <1.00E-15 |
| Unprotected:H:85:2070-Unprotected:C:45:2040 | 8.46E-03 | 8.44E-03 | 8.49E-03 | <1.00E-15 |
| Unprotected:H:45:2040-Protected:H:45:2040 | 1.07E-04 | 8.63E-05 | 1.28E-04 | <1.00E-15 |
| Protected:C:85:2040-Protected:H:45:2040 | 1.97E-04 | 1.75E-04 | 2.18E-04 | <1.00E-15 |
| Unprotected:C:85:2040-Protected:H:45:2040 | 1.02E-04 | 8.10E-05 | 1.23E-04 | <1.00E-15 |
| Protected:H:85:2040-Protected:H:45:2040 | 2.23E-03 | 2.21E-03 | 2.26E-03 | <1.00E-15 |
| Unprotected:H:85:2040-Protected:H:45:2040 | 2.41E-03 | 2.39E-03 | 2.43E-03 | <1.00E-15 |
| Protected:C:45:2070-Protected:H:45:2040 | 5.85E-04 | 5.64E-04 | 6.07E-04 | <1.00E-15 |
| Unprotected:C:45:2070-Protected:H:45:2040 | 4.37E-04 | 4.16E-04 | 4.58E-04 | <1.00E-15 |
| Protected:H:45:2070-Protected:H:45:2040 | 2.10E-03 | 2.08E-03 | 2.13E-03 | <1.00E-15 |
| Unprotected:H:45:2070-Protected:H:45:2040 | 2.28E-03 | 2.26E-03 | 2.30E-03 | <1.00E-15 |
| Protected:C:85:2070-Protected:H:45:2040 | 4.60E-03 | 4.58E-03 | 4.62E-03 | <1.00E-15 |
| Unprotected:C:85:2070-Protected:H:45:2040 | 4.36E-03 | 4.34E-03 | 4.38E-03 | <1.00E-15 |
| Protected:H:85:2070-Protected:H:45:2040 | 7.02E-03 | 7.00E-03 | 7.05E-03 | <1.00E-15 |
| Unprotected:H:85:2070-Protected:H:45:2040 | 6.95E-03 | 6.93E-03 | 6.97E-03 | <1.00E-15 |
| Protected:C:85:2040-Unprotected:H:45:2040 | 8.94E-05 | 6.84E-05 | 1.10E-04 | <1.00E-15 |
| Unprotected:C:85:2040-Unprotected:H:45:2040 | -5.30E-06 | -2.59E-05 | 1.53E-05 | **0.99996** |
| Protected:H:85:2040-Unprotected:H:45:2040 | 2.13E-03 | 2.11E-03 | 2.15E-03 | <1.00E-15 |
| Unprotected:H:85:2040-Unprotected:H:45:2040 | 2.30E-03 | 2.28E-03 | 2.32E-03 | <1.00E-15 |
| Protected:C:45:2070-Unprotected:H:45:2040 | 4.78E-04 | 4.57E-04 | 4.99E-04 | <1.00E-15 |
| Unprotected:C:45:2070-Unprotected:H:45:2040 | 3.30E-04 | 3.09E-04 | 3.50E-04 | <1.00E-15 |
| Protected:H:45:2070-Unprotected:H:45:2040 | 2.00E-03 | 1.98E-03 | 2.02E-03 | <1.00E-15 |
| Unprotected:H:45:2070-Unprotected:H:45:2040 | 2.18E-03 | 2.15E-03 | 2.20E-03 | <1.00E-15 |
| Protected:C:85:2070-Unprotected:H:45:2040 | 4.49E-03 | 4.47E-03 | 4.51E-03 | <1.00E-15 |
| Unprotected:C:85:2070-Unprotected:H:45:2040 | 4.25E-03 | 4.23E-03 | 4.27E-03 | <1.00E-15 |
| Protected:H:85:2070-Unprotected:H:45:2040 | 6.92E-03 | 6.90E-03 | 6.94E-03 | <1.00E-15 |
| Unprotected:H:85:2070-Unprotected:H:45:2040 | 6.84E-03 | 6.82E-03 | 6.86E-03 | <1.00E-15 |
| Unprotected:C:85:2040-Protected:C:85:2040 | -9.47E-05 | -1.16E-04 | -7.37E-05 | <1.00E-15 |
| Protected:H:85:2040-Protected:C:85:2040 | 2.04E-03 | 2.02E-03 | 2.06E-03 | <1.00E-15 |
| Unprotected:H:85:2040-Protected:C:85:2040 | 2.21E-03 | 2.19E-03 | 2.24E-03 | <1.00E-15 |
| Protected:C:45:2070-Protected:C:85:2040 | 3.89E-04 | 3.67E-04 | 4.10E-04 | <1.00E-15 |
| Unprotected:C:45:2070-Protected:C:85:2040 | 2.40E-04 | 2.19E-04 | 2.61E-04 | <1.00E-15 |
| Protected:H:45:2070-Protected:C:85:2040 | 1.91E-03 | 1.89E-03 | 1.93E-03 | <1.00E-15 |
| Unprotected:H:45:2070-Protected:C:85:2040 | 2.09E-03 | 2.06E-03 | 2.11E-03 | <1.00E-15 |
| Protected:C:85:2070-Protected:C:85:2040 | 4.40E-03 | 4.38E-03 | 4.42E-03 | <1.00E-15 |
| Unprotected:C:85:2070-Protected:C:85:2040 | 4.16E-03 | 4.14E-03 | 4.18E-03 | <1.00E-15 |
| Protected:H:85:2070-Protected:C:85:2040 | 6.83E-03 | 6.81E-03 | 6.85E-03 | <1.00E-15 |
| Unprotected:H:85:2070-Protected:C:85:2040 | 6.75E-03 | 6.73E-03 | 6.77E-03 | <1.00E-15 |
| Protected:H:85:2040-Unprotected:C:85:2040 | 2.13E-03 | 2.11E-03 | 2.15E-03 | <1.00E-15 |
| Unprotected:H:85:2040-Unprotected:C:85:2040 | 2.31E-03 | 2.29E-03 | 2.33E-03 | <1.00E-15 |
| Protected:C:45:2070-Unprotected:C:85:2040 | 4.83E-04 | 4.62E-04 | 5.04E-04 | <1.00E-15 |
| Unprotected:C:45:2070-Unprotected:C:85:2040 | 3.35E-04 | 3.14E-04 | 3.56E-04 | <1.00E-15 |
| Protected:H:45:2070-Unprotected:C:85:2040 | 2.00E-03 | 1.98E-03 | 2.02E-03 | <1.00E-15 |
| Unprotected:H:45:2070-Unprotected:C:85:2040 | 2.18E-03 | 2.16E-03 | 2.20E-03 | <1.00E-15 |
| Protected:C:85:2070-Unprotected:C:85:2040 | 4.50E-03 | 4.47E-03 | 4.52E-03 | <1.00E-15 |
| Unprotected:C:85:2070-Unprotected:C:85:2040 | 4.26E-03 | 4.24E-03 | 4.28E-03 | <1.00E-15 |
| Protected:H:85:2070-Unprotected:C:85:2040 | 6.92E-03 | 6.90E-03 | 6.94E-03 | <1.00E-15 |
| Unprotected:H:85:2070-Unprotected:C:85:2040 | 6.85E-03 | 6.83E-03 | 6.87E-03 | <1.00E-15 |
| Unprotected:H:85:2040-Protected:H:85:2040 | 1.77E-04 | 1.56E-04 | 1.98E-04 | <1.00E-15 |
| Protected:C:45:2070-Protected:H:85:2040 | -1.65E-03 | -1.67E-03 | -1.63E-03 | <1.00E-15 |
| Unprotected:C:45:2070-Protected:H:85:2040 | -1.80E-03 | -1.82E-03 | -1.78E-03 | <1.00E-15 |
| Protected:H:45:2070-Protected:H:85:2040 | -1.30E-04 | -1.51E-04 | -1.08E-04 | <1.00E-15 |
| Unprotected:H:45:2070-Protected:H:85:2040 | 4.91E-05 | 2.80E-05 | 7.01E-05 | <1.00E-15 |
| Protected:C:85:2070-Protected:H:85:2040 | 2.36E-03 | 2.34E-03 | 2.39E-03 | <1.00E-15 |
| Unprotected:C:85:2070-Protected:H:85:2040 | 2.13E-03 | 2.10E-03 | 2.15E-03 | <1.00E-15 |
| Protected:H:85:2070-Protected:H:85:2040 | 4.79E-03 | 4.77E-03 | 4.81E-03 | <1.00E-15 |
| Unprotected:H:85:2070-Protected:H:85:2040 | 4.71E-03 | 4.69E-03 | 4.74E-03 | <1.00E-15 |
| Protected:C:45:2070-Unprotected:H:85:2040 | -1.83E-03 | -1.85E-03 | -1.80E-03 | <1.00E-15 |
| Unprotected:C:45:2070-Unprotected:H:85:2040 | -1.97E-03 | -1.99E-03 | -1.95E-03 | <1.00E-15 |
| Protected:H:45:2070-Unprotected:H:85:2040 | -3.07E-04 | -3.28E-04 | -2.86E-04 | <1.00E-15 |
| Unprotected:H:45:2070-Unprotected:H:85:2040 | -1.28E-04 | -1.49E-04 | -1.08E-04 | <1.00E-15 |
| Protected:C:85:2070-Unprotected:H:85:2040 | 2.19E-03 | 2.17E-03 | 2.21E-03 | <1.00E-15 |
| Unprotected:C:85:2070-Unprotected:H:85:2040 | 1.95E-03 | 1.93E-03 | 1.97E-03 | <1.00E-15 |
| Protected:H:85:2070-Unprotected:H:85:2040 | 4.61E-03 | 4.59E-03 | 4.63E-03 | <1.00E-15 |
| Unprotected:H:85:2070-Unprotected:H:85:2040 | 4.54E-03 | 4.52E-03 | 4.56E-03 | <1.00E-15 |
| Unprotected:C:45:2070-Protected:C:45:2070 | -1.48E-04 | -1.69E-04 | -1.27E-04 | <1.00E-15 |
| Protected:H:45:2070-Protected:C:45:2070 | 1.52E-03 | 1.50E-03 | 1.54E-03 | <1.00E-15 |
| Unprotected:H:45:2070-Protected:C:45:2070 | 1.70E-03 | 1.68E-03 | 1.72E-03 | <1.00E-15 |
| Protected:C:85:2070-Protected:C:45:2070 | 4.01E-03 | 3.99E-03 | 4.03E-03 | <1.00E-15 |
| Unprotected:C:85:2070-Protected:C:45:2070 | 3.77E-03 | 3.75E-03 | 3.80E-03 | <1.00E-15 |
| Protected:H:85:2070-Protected:C:45:2070 | 6.44E-03 | 6.42E-03 | 6.46E-03 | <1.00E-15 |
| Unprotected:H:85:2070-Protected:C:45:2070 | 6.36E-03 | 6.34E-03 | 6.38E-03 | <1.00E-15 |
| Protected:H:45:2070-Unprotected:C:45:2070 | 1.67E-03 | 1.65E-03 | 1.69E-03 | <1.00E-15 |
| Unprotected:H:45:2070-Unprotected:C:45:2070 | 1.85E-03 | 1.83E-03 | 1.87E-03 | <1.00E-15 |
| Protected:C:85:2070-Unprotected:C:45:2070 | 4.16E-03 | 4.14E-03 | 4.18E-03 | <1.00E-15 |
| Unprotected:C:85:2070-Unprotected:C:45:2070 | 3.92E-03 | 3.90E-03 | 3.94E-03 | <1.00E-15 |
| Protected:H:85:2070-Unprotected:C:45:2070 | 6.59E-03 | 6.57E-03 | 6.61E-03 | <1.00E-15 |
| Unprotected:H:85:2070-Unprotected:C:45:2070 | 6.51E-03 | 6.49E-03 | 6.53E-03 | <1.00E-15 |
| Unprotected:H:45:2070-Protected:H:45:2070 | 1.79E-04 | 1.58E-04 | 2.00E-04 | <1.00E-15 |
| Protected:C:85:2070-Protected:H:45:2070 | 2.49E-03 | 2.47E-03 | 2.51E-03 | <1.00E-15 |
| Unprotected:C:85:2070-Protected:H:45:2070 | 2.26E-03 | 2.23E-03 | 2.28E-03 | <1.00E-15 |
| Protected:H:85:2070-Protected:H:45:2070 | 4.92E-03 | 4.90E-03 | 4.94E-03 | <1.00E-15 |
| Unprotected:H:85:2070-Protected:H:45:2070 | 4.84E-03 | 4.82E-03 | 4.86E-03 | <1.00E-15 |
| Protected:C:85:2070-Unprotected:H:45:2070 | 2.31E-03 | 2.29E-03 | 2.34E-03 | <1.00E-15 |
| Unprotected:C:85:2070-Unprotected:H:45:2070 | 2.08E-03 | 2.06E-03 | 2.10E-03 | <1.00E-15 |
| Protected:H:85:2070-Unprotected:H:45:2070 | 4.74E-03 | 4.72E-03 | 4.76E-03 | <1.00E-15 |
| Unprotected:H:85:2070-Unprotected:H:45:2070 | 4.67E-03 | 4.64E-03 | 4.69E-03 | <1.00E-15 |
| Unprotected:C:85:2070-Protected:C:85:2070 | -2.38E-04 | -2.59E-04 | -2.17E-04 | <1.00E-15 |
| Protected:H:85:2070-Protected:C:85:2070 | 2.43E-03 | 2.40E-03 | 2.45E-03 | <1.00E-15 |
| Unprotected:H:85:2070-Protected:C:85:2070 | 2.35E-03 | 2.33E-03 | 2.37E-03 | <1.00E-15 |
| Protected:H:85:2070-Unprotected:C:85:2070 | 2.66E-03 | 2.64E-03 | 2.69E-03 | <1.00E-15 |
| Unprotected:H:85:2070-Unprotected:C:85:2070 | 2.59E-03 | 2.57E-03 | 2.61E-03 | <1.00E-15 |
| Unprotected:H:85:2070-Protected:H:85:2070 | -7.59E-05 | -9.70E-05 | -5.49E-05 | <1.00E-15 |

**
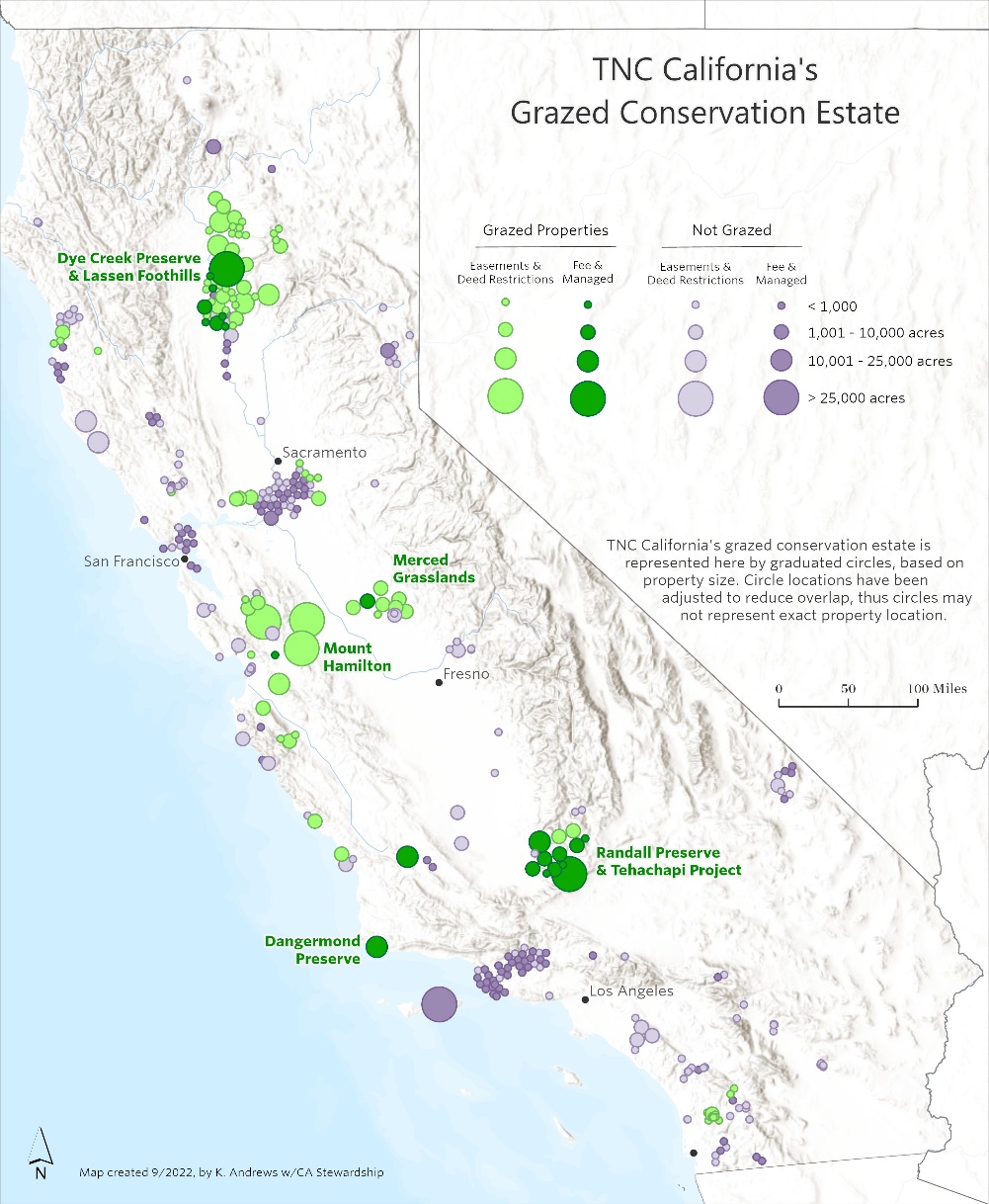
**

**Supplementary Figure 1.** The Nature Conservancy’s conservation estate across California. Each circle represents a managed property, with the size of the circle corresponding to the size of the preserve.


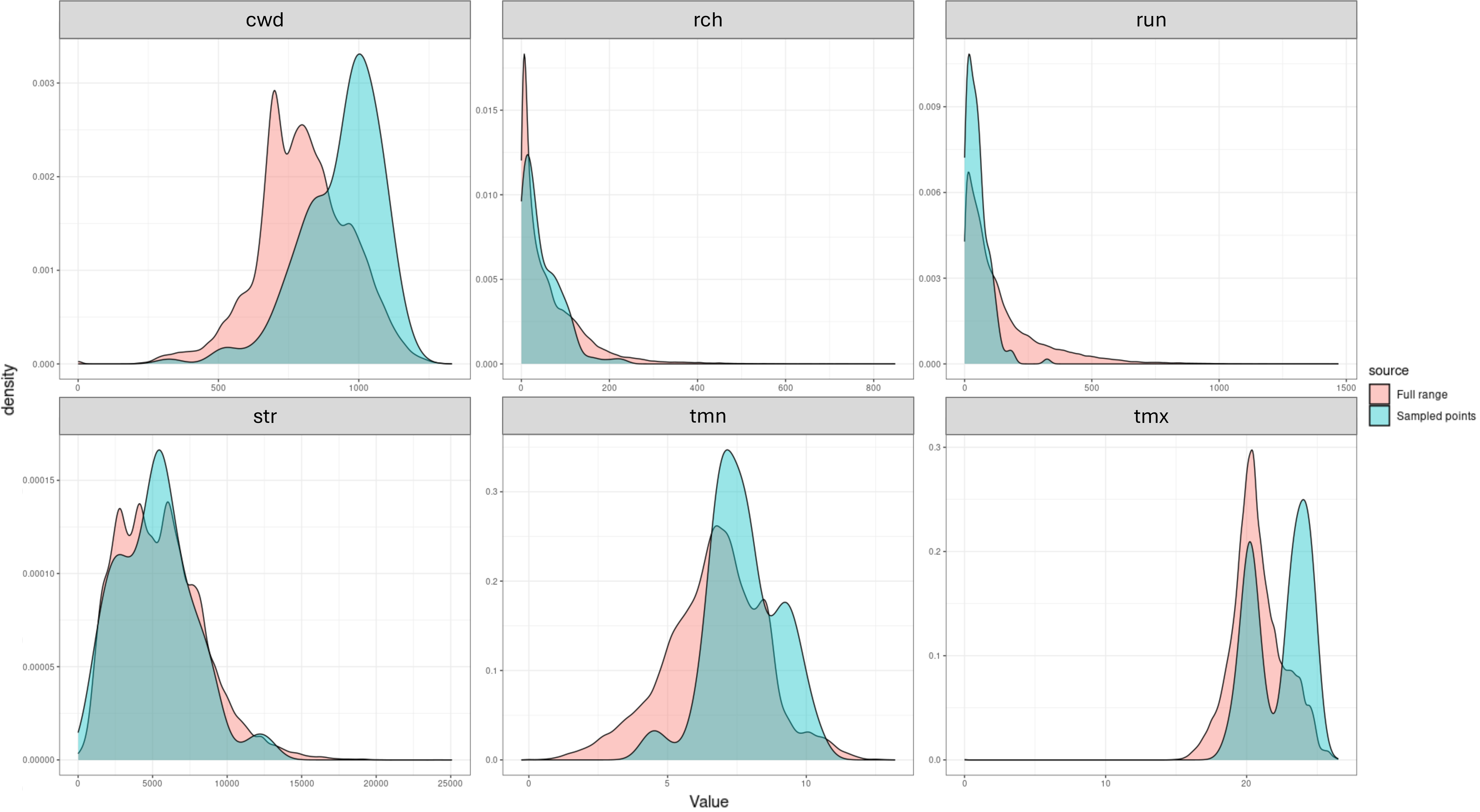


**Supplementary Figure 2.** Environmental gradients captured by our sampling design (blue) vs the entire species distribution of coast live oak (red).


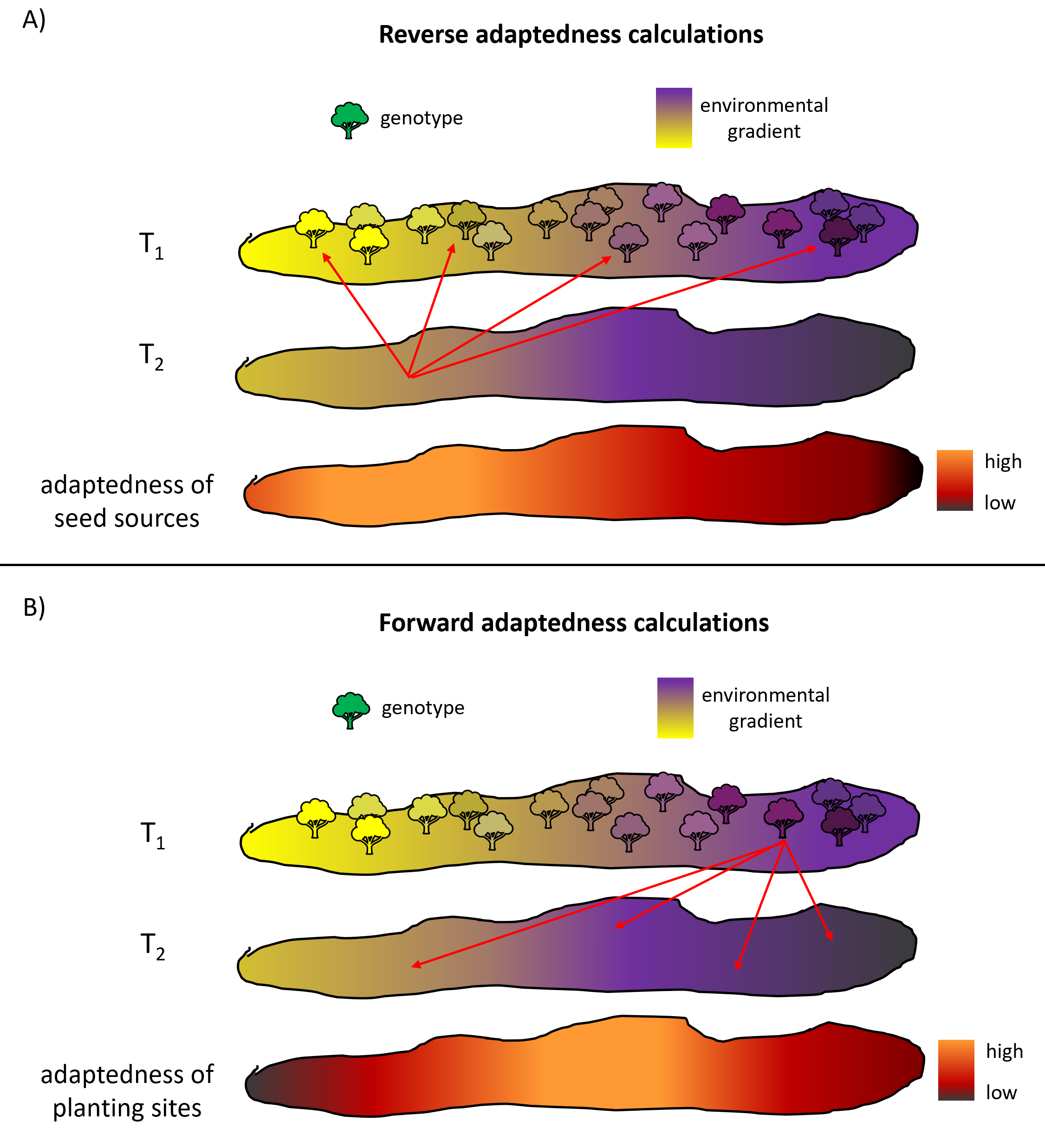


**Supplementary Figure 3.** Graphical illustration of how adaptedness after seed transfer is calculated. A) To find potential seed sources that would be preadapted to a future planting site, reverse adaptedness is calculated between the adaptive variation needed in the future climate (T_2_) of a recipient cell and the adaptive variation currently present (T_1_) in potential donor cells. Adaptedness values can then be mapped to show stands’ adaptedness if transferred to a given planting site. B) To find potential future planting sites that current seed sources would be preadapted to, forward adaptedness is calculated between the adaptive variation currently present (T_1_) in donor cells and the adaptive variation needed in the future climate (T_2_) of a potential recipient cells. Adaptedness values can then be mapped to show stands’ adaptedness if given seeds were transferred into the site.


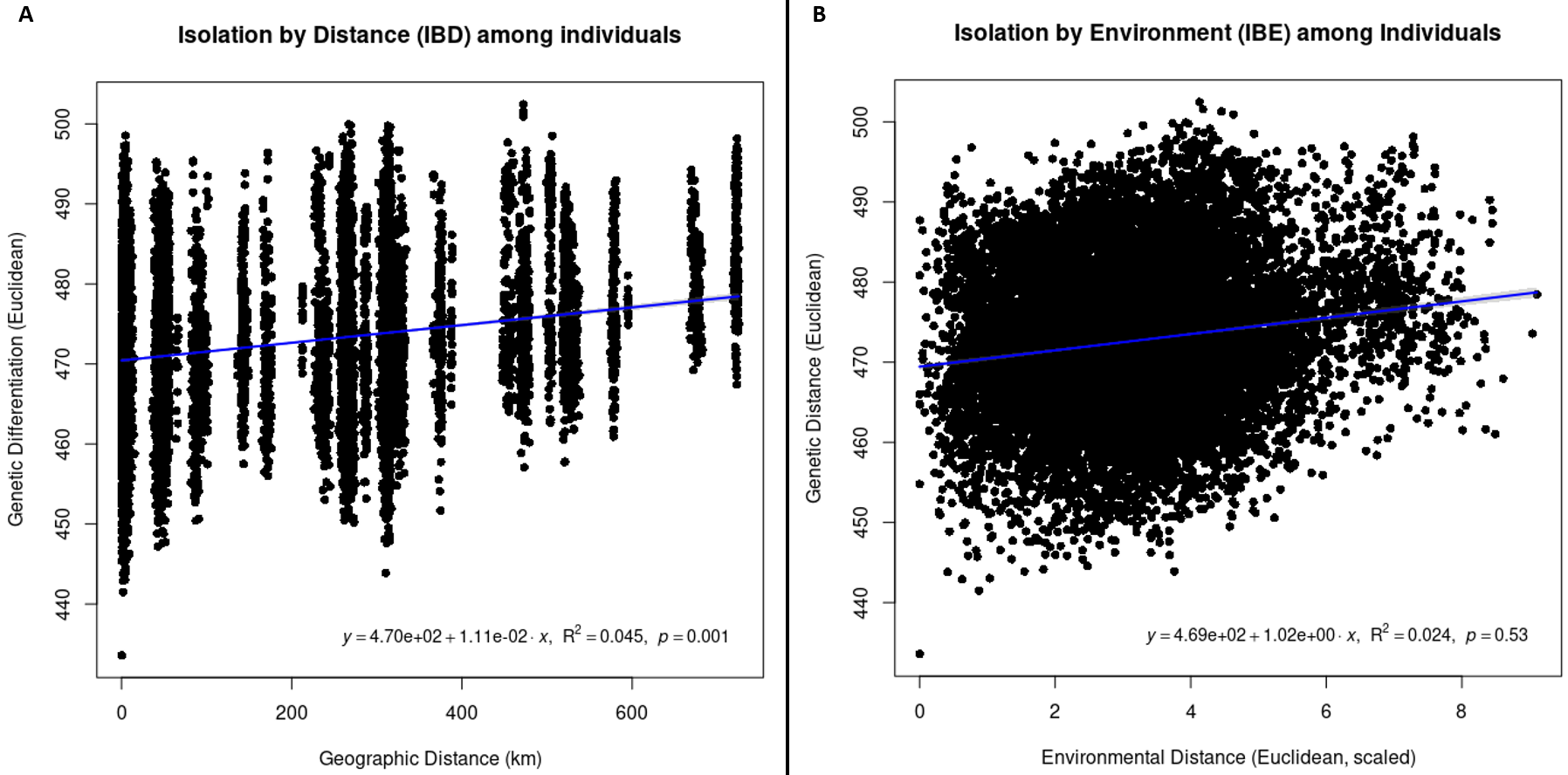


**Supplementary Figure 4.** A) Isolation by distance results showing geographic distance in kilometers and genetic distance (calculated as the Euclidean distance between individual genotypes) with each dot representing a pairwise comparison between individual genotypes. The trend was significant (p = 0.001), suggesting that geographic distance is positively correlated with genetic distance. B) Isolation by environment accounting for geography results showing environmental distance (calculated as the Euclidean distances of scaled mean environmental variables at each individual’s location) and genetic distance (calculated as the Euclidean distance between individual genotypes) with each dot representing a pairwise comparison between individuals. The trend was not significant (p = 0.53), suggesting that environmental distance is not significantly correlated with genetic distance.


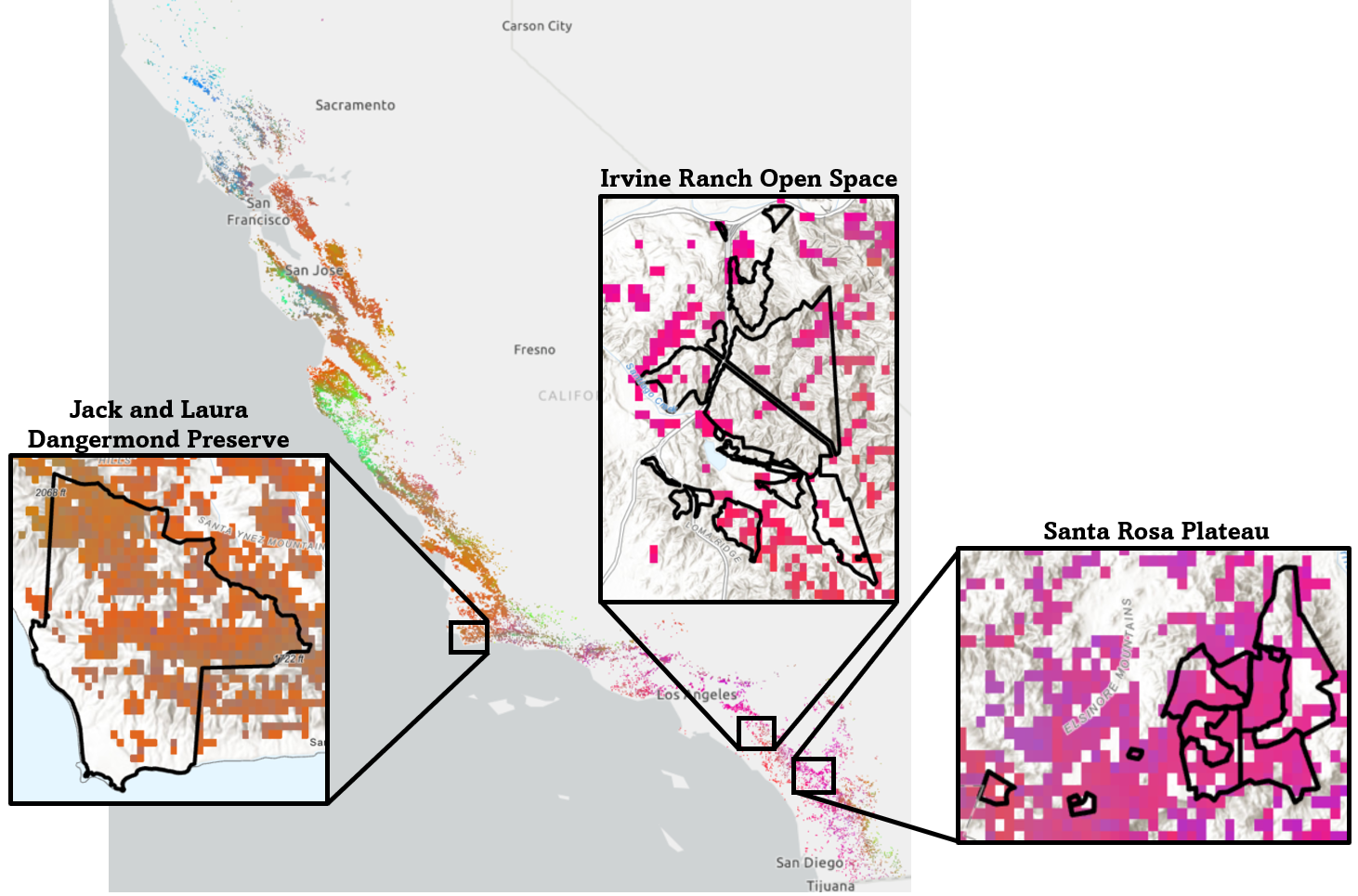


**Supplementary Figure 5.** Genomic turnover showing adaptive composition interpolated across coast live oak’s range by gradient forest models. Larger differences in colors indicate larger differences in adaptive composition. Overlaid boxes contain enlarged adaptive composition of the three TNC focal sites.


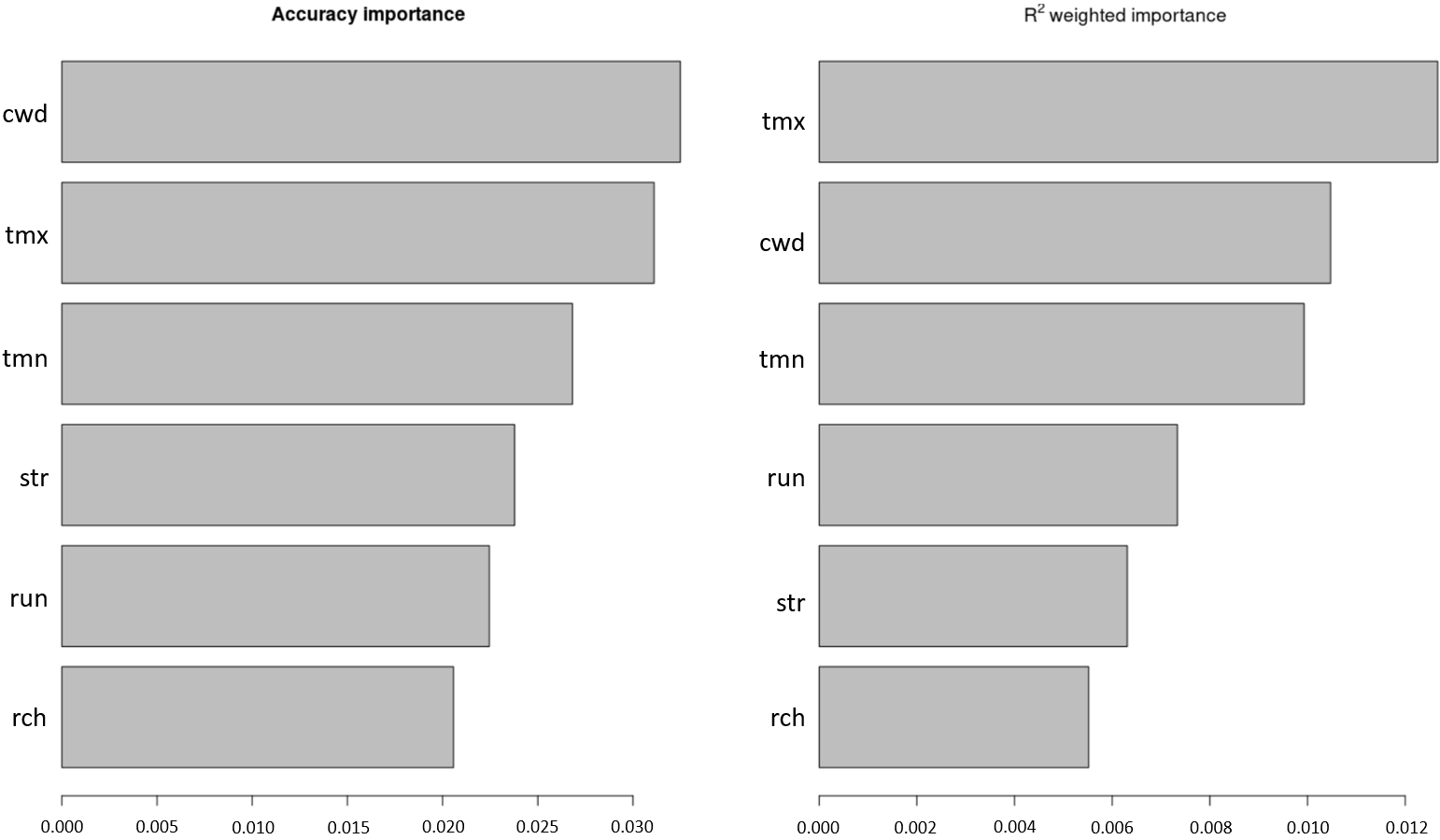


**Supplementary Figure 6.** Importance of bioclimatic variables in the gradient forest models. The left graph shows the unweighted average of split importances across loci, or how often a predictor variable was used to split regression trees, while the right graph shows the split importance weighted by the variance explained for each locus. SNPs with non-zero R^2^ in the GF model (942 SNPs) had predictive power and were thus used by the model in the turnover and offset predictions.


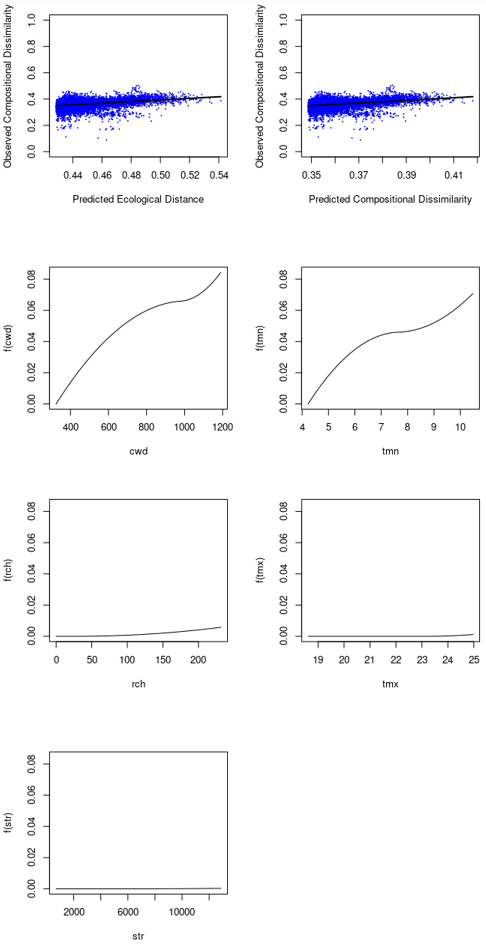


**Supplementary Figure 7.** Importance of bioclimatic variables in the generalized dissimilarity models, with each as a function of variation explained. Climate water deficit (cwd is the most important predictor of genetic dissimilarity, followed by minimum temperature (tmn), soil recharge (rch), maximum temperature (tmax), and soil storage (str). The first graph includes environmental distance by observed genetic distance, with the positive slope indicating isolation by environment, which is expected when examining genotype-environment associations. Note that runoff is not pictured because the model did not find it explained a significant amount of variation.


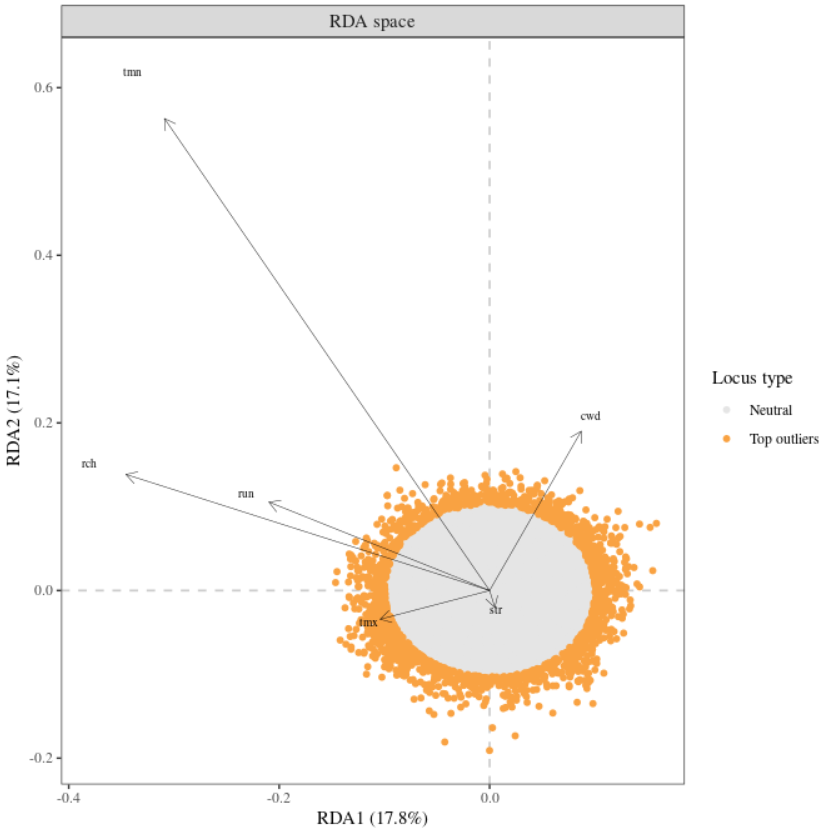


**Supplementary Figure 8.** Distribution of genomic variation in RDA space using all loci, with RDA1 accounting for 17.8% of variation and RDA2 accounting for 17.1%. The 1,741 outlier loci above the Bonferroni-corrected significance threshold (α=0.01/*n*) are colored orange, while the other 946,645 loci are colored grey. The strength of each bioclimatic variable on variation is represented by the black loading arrows, with longer arrows representing more importance in explaining variation in the direction they are pointing.

**
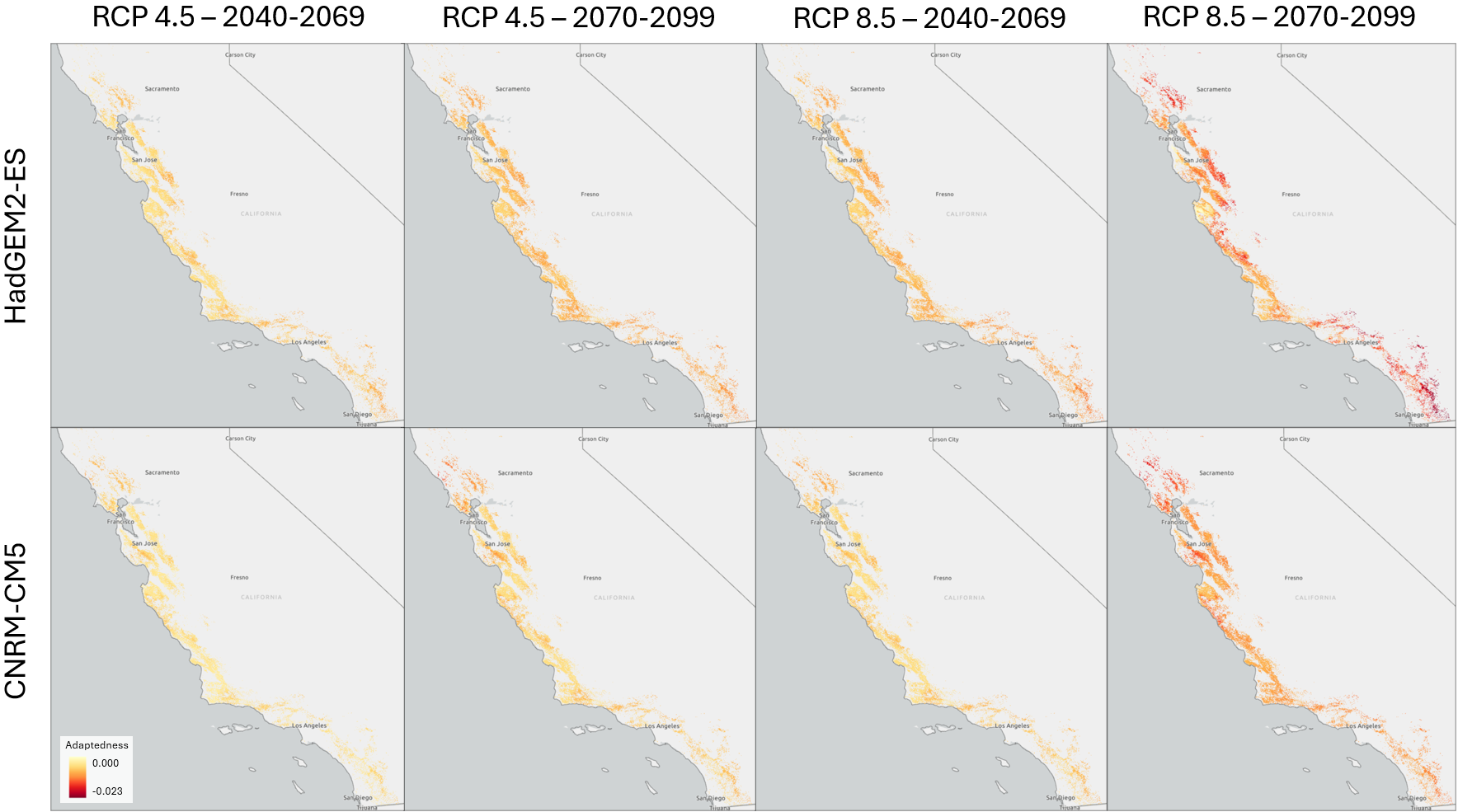
Supplementary Figure 9**. Adaptedness predictions using gradient forest for RCP 4.5 and 8.5, climate models HadGEM2-ES and CNRM-CM5, and 30-year average time periods 2040-2069 and 2070-2099. Lighter colors indicate higher adaptedness values.


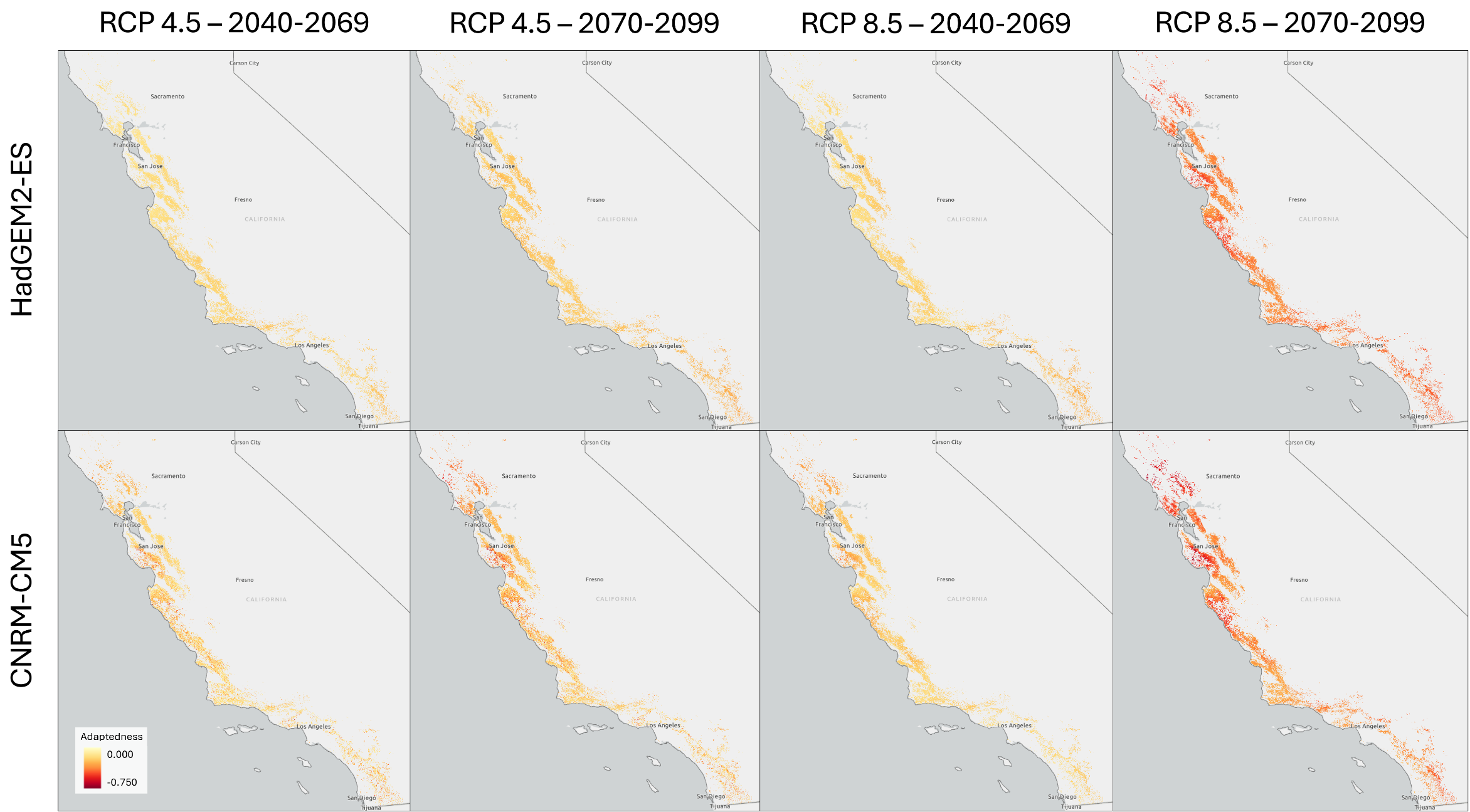
**Supplementary Figure** **10.** Adaptedness predictions using redundancy analysis (RDA) for RCP 4.5 and 8.5, climate models HadGEM2-ES and CNRM-CM5, and 30-year average time periods 2040-2069 and 2070-2099. Lighter colors indicate higher adaptedness values.


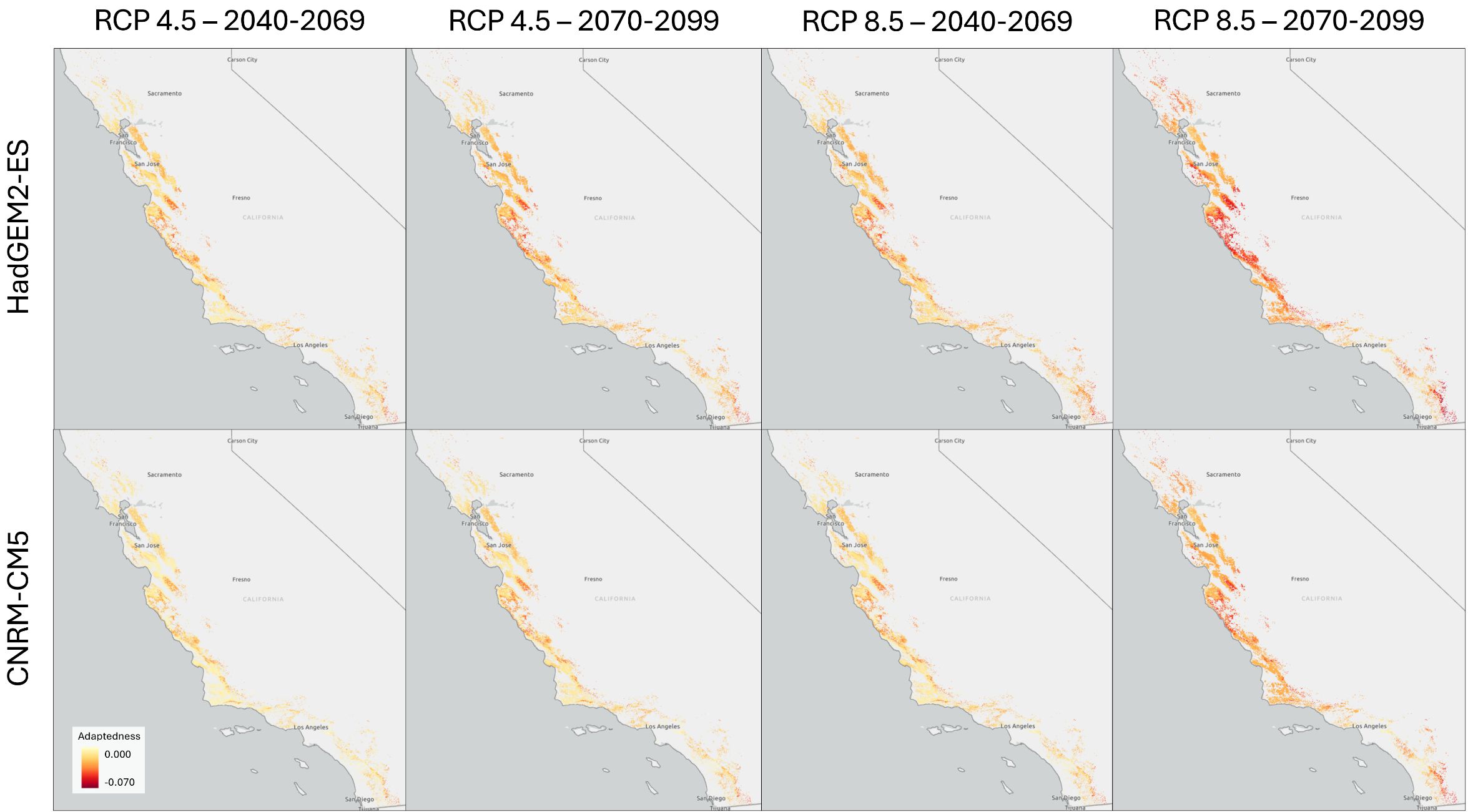
**Supplementary Figure** **11.** Adaptedness predictions using generalized dissimilarity models (GDM) for RCP 4.5 and 8.5, climate models HadGEM2-ES and CNRM-CM5, and 30-year average time periods 2040-2069 and 2070-2099. Lighter colors indicate higher adaptedness values.


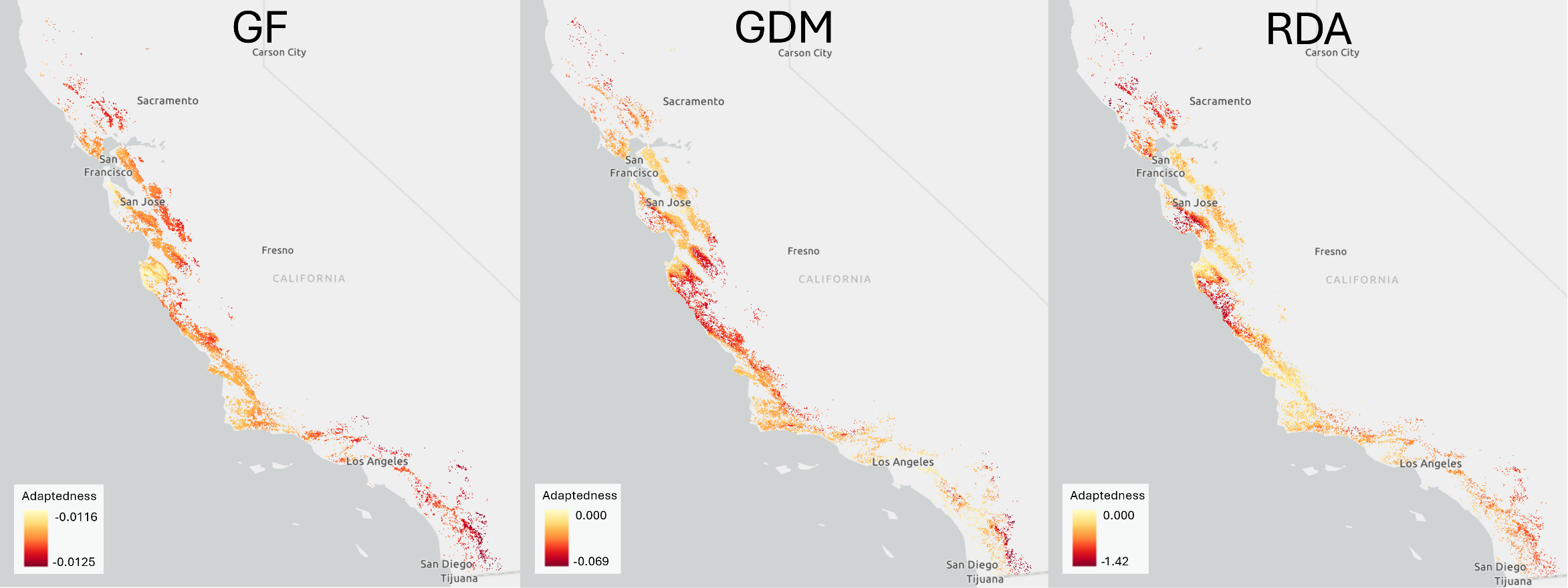
**Supplementary Figure** **12.** Adaptedness predictions of the three methods (gradient forest = GF, generalized dissimilarity models = GDM, redundancy analysis = RDA) for HadGEM2-ES RCP 8.5 2070-2099. Lighter colors indicate higher adaptedness values. Adaptedness values are not directly comparable among methods, but patterns of highest and lowest adaptedness are.


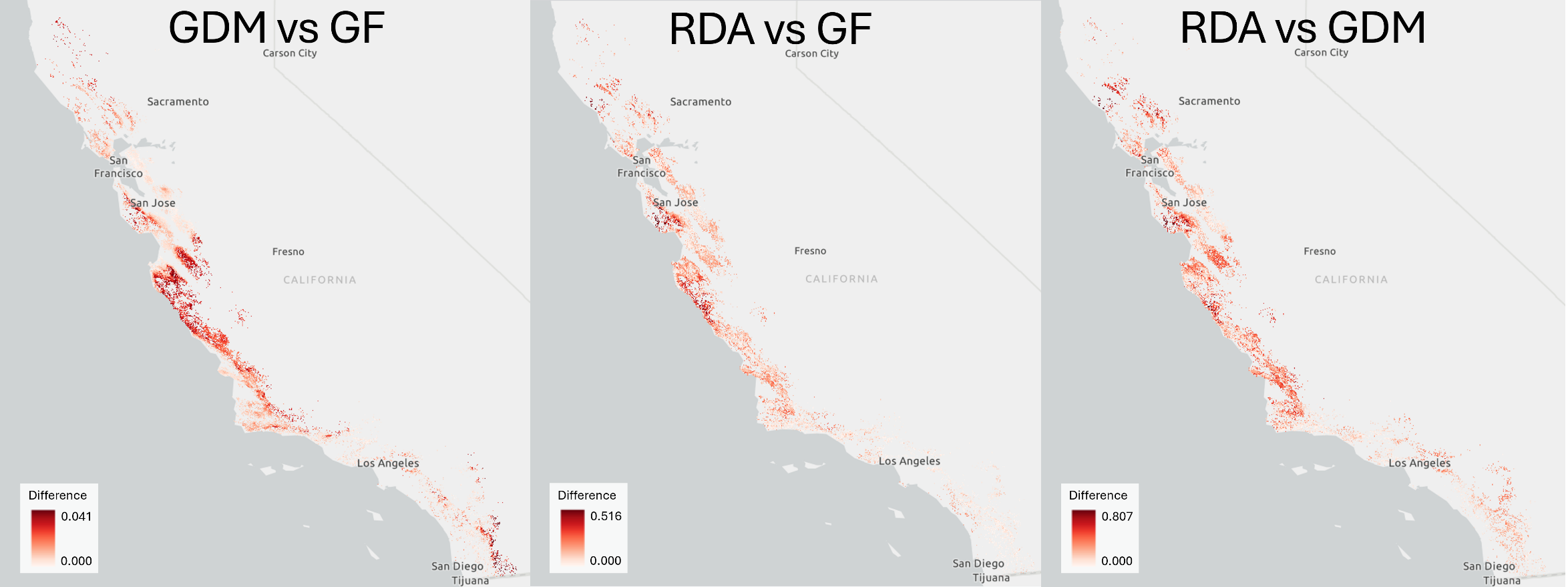
**Supplementary Figure** **13.** Procrustes residuals from pairwise adaptedness method comparison (GF = gradient forest, GDM = generalized dissimilarity model, RDA = redundancy analysis) for HadGEM2-ES RCP 8.5 2070-2099. Darker colors indicate larger differences between methods. Values are not directly comparable among analyses but patterns of largest and smallest differences are.


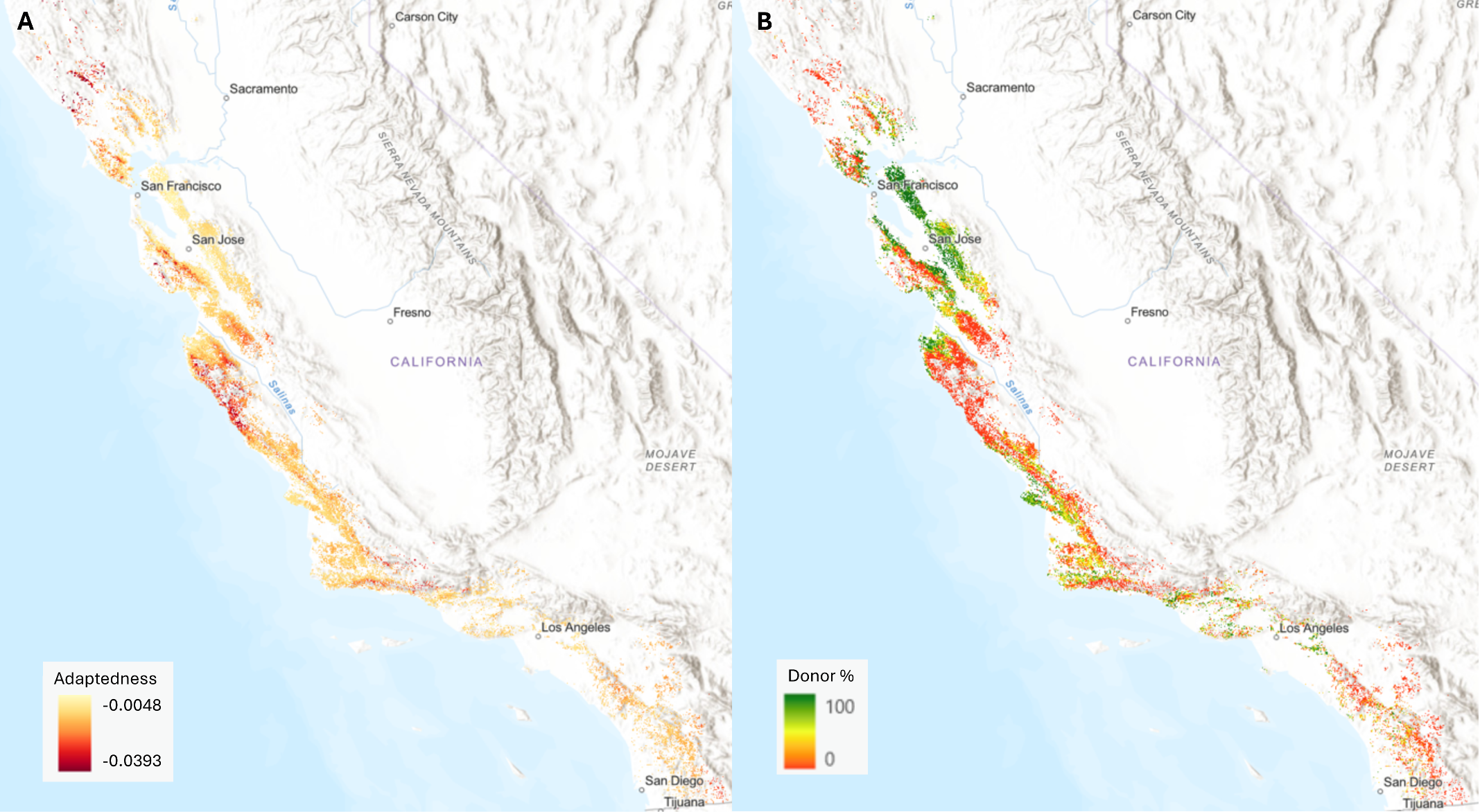
**Supplementary Figure** **14**. A) Reverse adaptedness values from the entire coast live oak range to the Jack and Laura Dangermond Preserve modeled in HadGEM-ES2 RCP 8.5 2070-2099, with lighter colors indicating higher average adaptedness of Jack and Laura Dangermond Preserve if the grid cell was used as a seed source. B) Seed source priority from the entire coast live oak range to the Jack and Laura Dangermond Preserve modeled in HadGEM-ES2 RCP 8.5 2070-2099, with greener colors indicating a higher percentage of Jack and Laura Dangermond Preserve that would have increased adaptedness if the grid cell was used as a seed source.


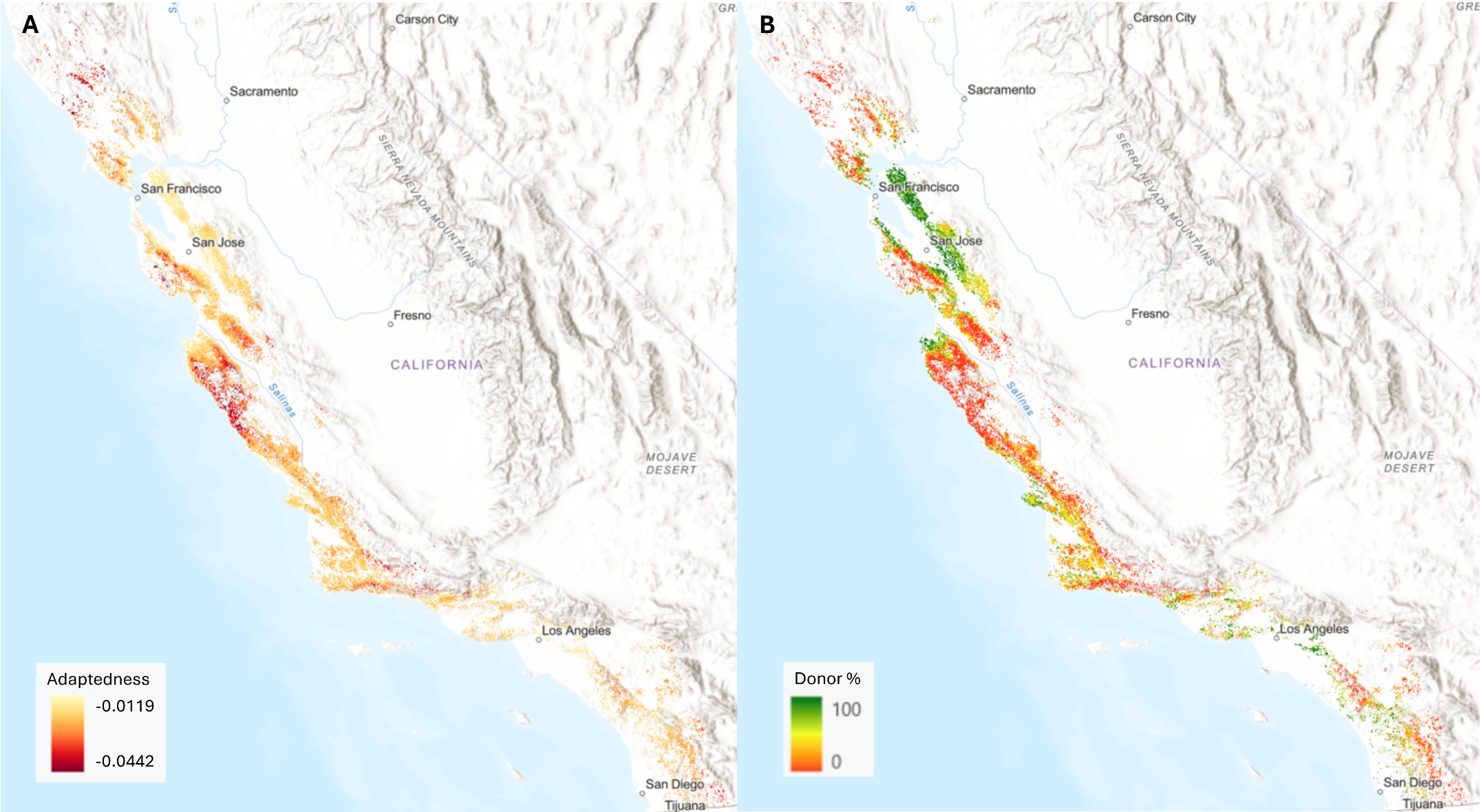
**Supplementary Figure** **15.** A) Reverse adaptedness values from the entire coast live oak range to Irvine Ranch Open Space modeled in HadGEM-ES2 RCP 8.5 2070-2099, with lighter colors indicating higher average adaptedness of Irvine Ranch Open Space if the grid cell was used as a seed source. B) Seed source priority from the entire coast live oak range to Irvine Ranch Open Space modeled in HadGEM-ES2 RCP 8.5 2070-2099, with greener colors indicating a higher percentage of Irvine Ranch Open Space that would have increased adaptedness if the grid cell was used as a seed source.


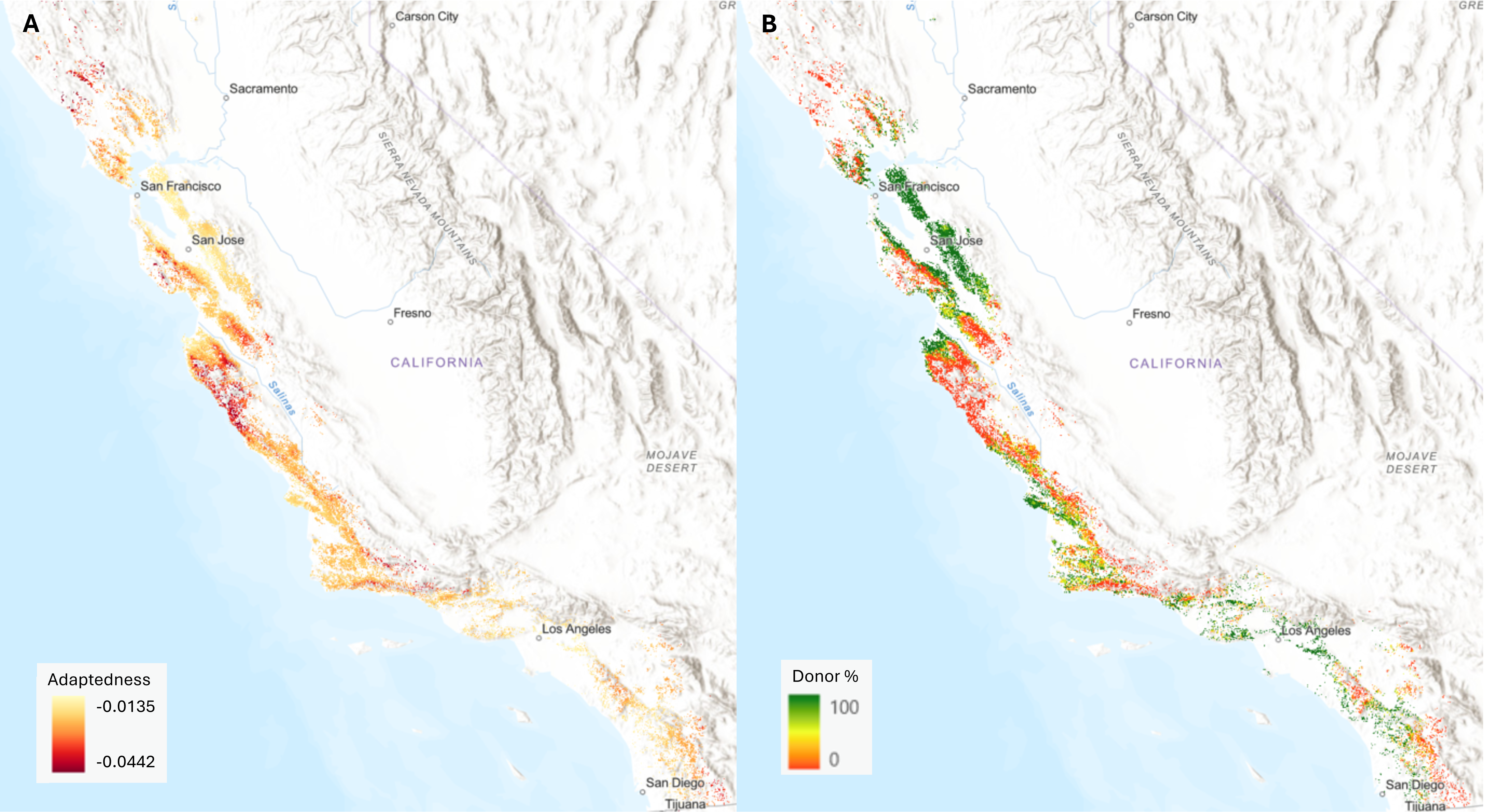
 **Supplementary Figure** **16.** A) Reverse adaptedness values from the entire coast live oak range to Santa Rose Plateau modeled in HadGEM-ES2 RCP 8.5 2070-2099, with lighter colors indicating higher average adaptedness of Santa Rosa Plateau if the grid cell was used as a seed source. B) Seed source priority from the entire coast live oak range to Santa Rosa Plateau modeled in HadGEM-ES2 RCP 8.5 2070-2099, with greener colors indicating a higher percentage of Santa Rosa Plateau that would have increased adaptedness if the grid cell was used as a seed source.


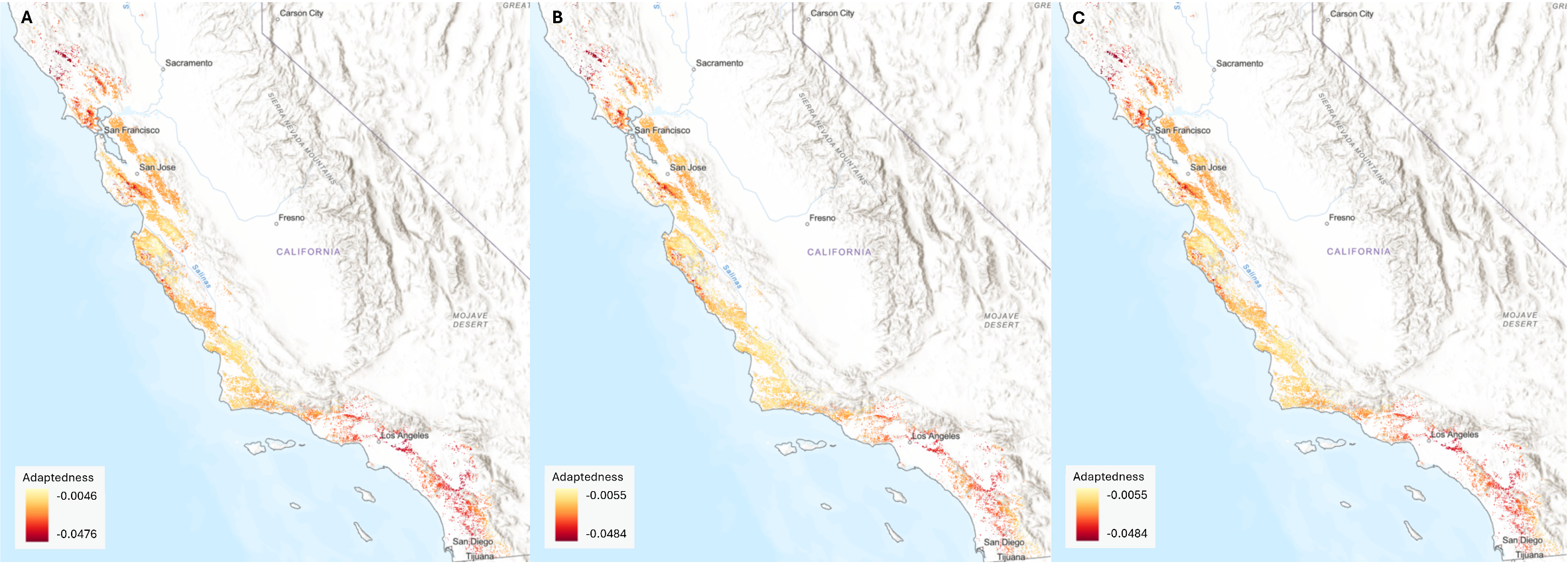
**Supplementary Figure** **17.** Forward adaptedness values from each focal site (A = Jack and Laura Dangermond Preserve, B = Irvine Ranch Open Space, C = Santa Rosa Plateau) to the entire coast live oak range modeled in HadGEM-ES2 RCP 8.5 2070-2099, with lighter colors indicating higher average adaptedness of each focal site if the grid cell was used as a planting location.


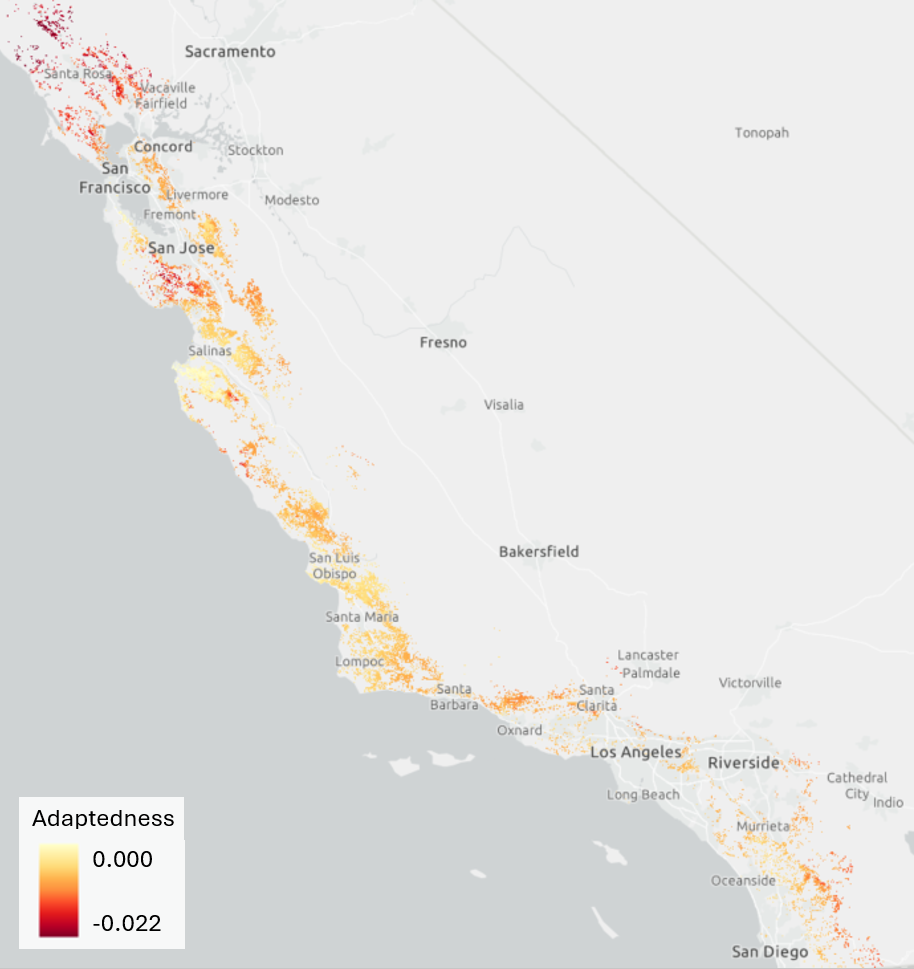


**Supplementary Figure 18.** Adaptedness predictions within unprotected lands for climate model CNRM-CM5, RCP 8.5, 2070-2099. Lighter colors indicate higher adaptedness values and thus less predicted maladaptation to future climate.


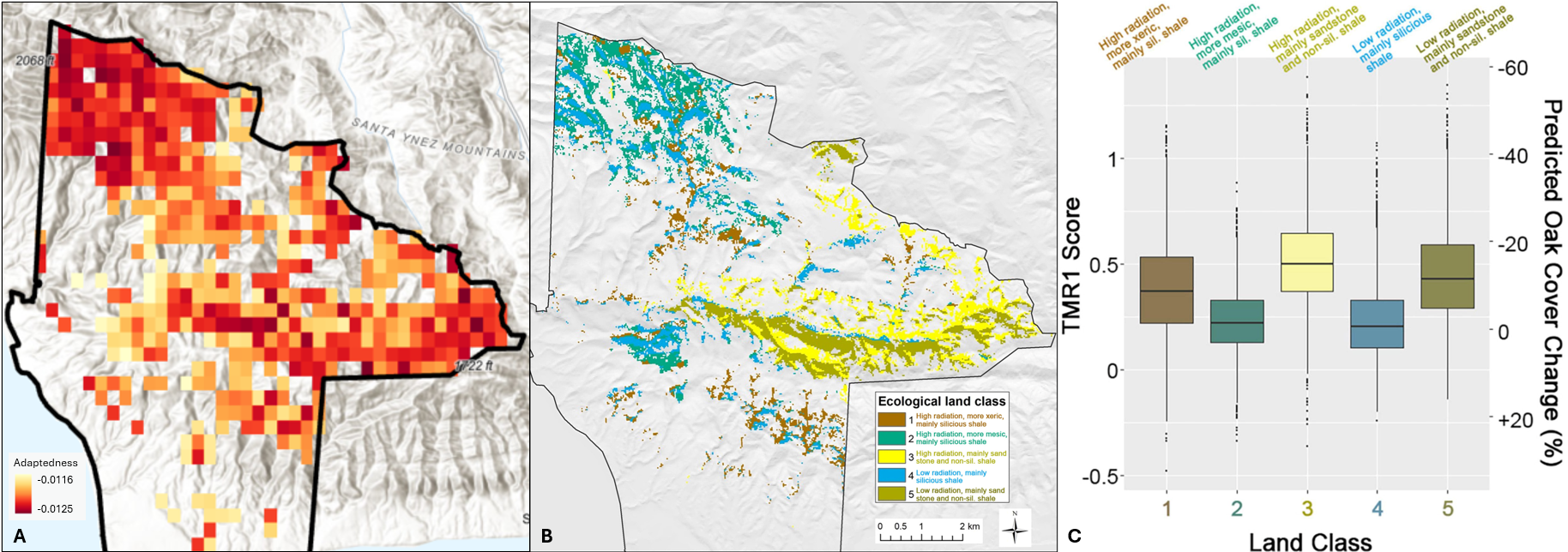


**Supplementary Figure 19.** Side-by-side visual comparison of adaptedness results (A) within Jack and Laura Dangermond Preserve modeled in this study, with the land classes (B) and corresponding oak cover change (C) predicted by Sousa et al. (2022). It is important to note the scale of adaptedness values in A, which are relative to the Jack and Laura Dangermond Preserve, not to the entire coast live oak range as shown in Fig. 3. This was done to emphasize the differences in adaptedness values within the preserve.

**
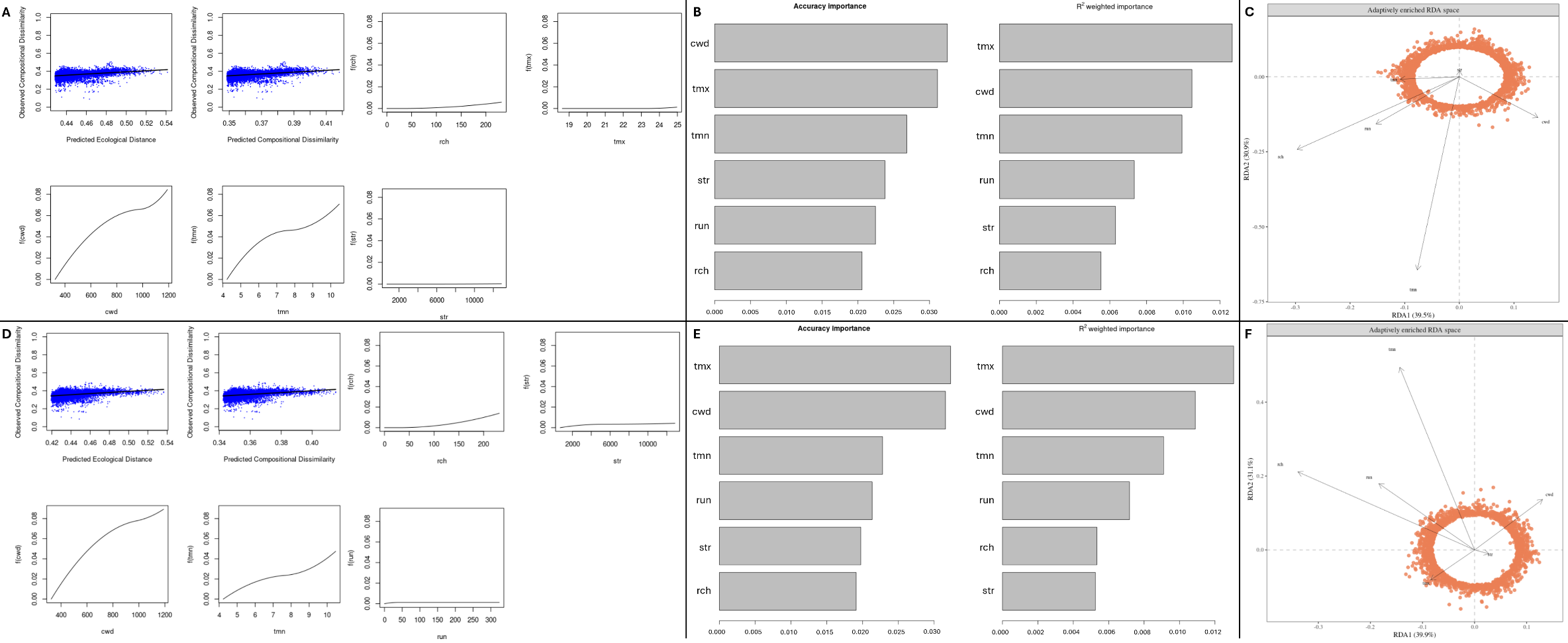
****Supplementary Figure 20.** Importance of climate variables in explaining putatively adaptive variation when including PC1 (A-C) and not (D-F) in the pRDA to find outlier loci. A and D show generalized dissimilarity model (GDM) variable importance, B and E show gradient forest (GF) variable importance, and C and F show redundancy analysis (RDA) variable importance.

**References**

Capblancq, T., and B. R. Forester. 2021. “Redundancy analysis: A Swiss Army Knife for landscape genomics.” Methods in Ecology and Evolution, no. 12: 2298-2309.

Ellis, N., S. J. Smith, and C. R. Pitcher. 2012. “Gradient Forests: Calculating Importance Gradients on Physical Predictors.” Ecology 93, no. 1: 156–168.

ESRI 2025. ArcGIS Pro 3.6.0. Redlands, CA: Environmental Systems Research Institute.

Ferrier, S., G. Manion, J. Elith, and K. Richardson. 2007. “Using Generalized Dissimilarity Modelling to Analyse and Predict Patterns of Beta Diversity in Regional Biodiversity Assessment.” Diversity and Distributions 13, no. 3: 252–264.

Fitzpatrick, M. C., and S. R. Keller. 2015. “Ecological Genomics Meets Community‐Level Modelling of Biodiversity: Mapping the Genomic Landscape of Current and Future Environmental Adaptation.” Ecology Letters 18, no. 1: 1–16..

Fitzpatrick, M. C., K. Mokany, G. Manion, D. Nieto‐Lugilde, and S. Ferrier. 2024. “gdm: Generalized Dissimilarity Modeling. R Package Version 1.6.”

Gougherty, A. V., S. R. Keller, and M. C. Fitzpatrick. 2021. “Maladaptation, Migration and Extirpation Fuel Climate Change Risk in a Forest Tree Species.” Nature Climate Change 11, no. 2: 166–171.

Stern, M. A., L. E. Flint, A. L. Flint, and W. A. Seymour. 2024. “Future Climate and Hydrology From Twenty Localized Constructed Analog (LOCA) Scenarios and the Basin Characterization Model (BCMv8) (ver. 1.1, November 2024): U.S. Geological Survey Data Release.”
